# SARS-CoV-2 spike conformation determines plasma neutralizing activity elicited by a wide panel of human vaccines

**DOI:** 10.1126/sciimmunol.adf1421

**Published:** 2022-11-10

**Authors:** John E. Bowen, Young-Jun Park, Cameron Stewart, Jack T. Brown, William K. Sharkey, Alexandra C. Walls, Anshu Joshi, Kaitlin R. Sprouse, Matthew McCallum, M. Alejandra Tortorici, Nicholas M. Franko, Jennifer K. Logue, Ignacio G. Mazzitelli, Annalee W. Nguyen, Rui P. Silva, Yimin Huang, Jun Siong Low, Josipa Jerak, Sasha W Tiles, Kumail Ahmed, Asefa Shariq, Jennifer M. Dan, Zeli Zhang, Daniela Weiskopf, Alessandro Sette, Gyorgy Snell, Christine M. Posavad, Najeeha Talat Iqbal, Jorge Geffner, Alessandra Bandera, Andrea Gori, Federica Sallusto, Jennifer A. Maynard, Shane Crotty, Wesley C. Van Voorhis, Carlos Simmerling, Renata Grifantini, Helen Y. Chu, Davide Corti, David Veesler

**Affiliations:** ^1^ Department of Biochemistry, University of Washington, Seattle, WA 98195, USA.; ^2^ Howard Hughes Medical Institute, University of Washington, Seattle, WA 98195, USA.; ^3^ Division of Allergy and Infectious Diseases, University of Washington, Seattle, WA 98195, USA.; ^4^ Instituto de Investigaciones Biomédicas en Retrovirus y SIDA (INBIRS), Facultad de Medicina, Buenos Aires C1121ABG, Argentina.; ^5^ McKetta Department of Chemical Engineering, The University of Texas at Austin, Austin, TX.; ^6^ Institute for Research in Biomedicine, Università della Svizzera Italiana, 6500 Bellinzona, Switzerland.; ^7^ Center for Emerging and Re-emerging Infectious Diseases, Division of Allergy and Infectious Diseases, Department of Medicine, University of Washington School of Medicine, Seattle, WA 98195, USA.; ^8^ Department of Paediatrics and Child Health, and Biological & Biomedical Sciences, Aga Khan University, Karachi 74800, Pakistan.; ^9^ Center for Infectious Disease and Vaccine Research, La Jolla Institute for Immunology, La Jolla, CA 92037, USA.; ^10^ Department of Medicine, Division of Infectious Diseases and Global Public Health, University of California, San Diego, La Jolla, CA UC92037, USA.; ^11^ Vir Biotechnology, San Francisco, CA 94158, USA.; ^12^ Vaccine and Infectious Disease Division, Fred Hutchinson Cancer Center, Seattle, WA, USA.; ^13^ Infectious Diseases Unit, Fondazione IRCCS Ca’ Granda Ospedale Maggiore Policlinico, 20122 Milan, Italy.; ^14^ Department of Chemistry, Stony Brook University, Stony Brook, New York 11794, United States.; ^15^ Laufer Center for Physical and Quantitative Biology, Stony Brook University, Stony Brook, New York 11794, United States.; ^16^ INGM, Istituto Nazionale Genetica Molecolare “Romeo ed Enrica Invernizzi”, 20122 Milan, Italy.; ^17^ Humabs Biomed SA, a subsidiary of Vir Biotechnology, 6500 Bellinzona, Switzerland.

## Abstract

Numerous safe and effective COVID-19 vaccines have been developed worldwide that utilize various delivery technologies and engineering strategies. We show here that vaccines containing prefusion-stabilizing S mutations elicit antibody responses in humans with enhanced recognition of S and the S_1_ subunit relative to postfusion S, as compared to vaccines lacking these mutations or natural infection. Prefusion S and S_1_ antibody binding titers positively and equivalently correlated with neutralizing activity and depletion of S_1_-directed antibodies completely abrogated plasma neutralizing activity. We show that neutralizing activity is almost entirely directed to the S_1_ subunit and that variant cross-neutralization is mediated solely by RBD-specific antibodies. Our data provide a quantitative framework for guiding future S engineering efforts to develop vaccines with higher resilience to the emergence of variants than current technologies.

## Teaser

SARS-CoV-2 S1 accounts for all vaccine-elicited plasma neutralization and cross-neutralization is mediated by RBD-specific antibodies.

## INTRODUCTION

The SARS-CoV-2 spike (S) glycoprotein promotes viral entry into host cells and is the main target of neutralizing antibodies (*1*, *2*). S comprises two functional subunits, designated S_1_ and S_2_, that interact non-covalently after furin cleavage during synthesis (*1*, *3*, *4*). The receptor-binding domain (RBD), which engages the ACE2 receptor (*1*, *3*, *5*–*7*), and the N-terminal domain (NTD) that recognizes attachment factors (*8*–*10*) are components of the S_1_ subunit. The S_2_ subunit contains the fusion machinery and undergoes large-scale structural rearrangements from a high-energy spring-loaded prefusion conformation to a postfusion state, driving fusion of the virus and host membranes and initiating infection (*11*–*13*). Antibodies that bind to specific sites on the RBD (*14*–*24*), the NTD (*25*–*29*), or the fusion machinery (*30*–*36*) neutralize SARS-CoV-2 and serum neutralizing antibody titers are a correlate of protection (*37*–*43*).

As of October 2022, more than 12.8 billion COVID-19 vaccine doses have been administered worldwide. Moderna/NIAID mRNA-1273 and Pfizer/BioNTech BNT162b2 were conceived as two-dose vaccines based on an mRNA encoding the full-length prefusion-stabilized ‘2P’ S glycoprotein encapsulated in a lipid nanoparticle (*44*–*46*). Novavax NVX-CoV2373 is a prefusion-stabilized ‘2P’ S protein-subunit vaccine with a mutated furin cleavage site and formulated with a saponin-based matrix M adjuvant (*47*) whereas AstraZeneca/Oxford AZD1222, Gamaleya Research Institute Sputnik V, and Janssen Ad26.COV2.S are replication-defective adenoviral-vectored vaccines encoding for the full-length S glycoprotein. Only Ad26.COV2.S encodes for a prefusion-stabilized S with the ‘2P’ mutations and mutated furin cleavage site (*48*) whereas the other two vaccines lack these modifications. The adenoviral vectors used are chimpanzee AdY25 for AZD1222 (*49*) and Ad26 (prime)/Ad5 (boost) for Sputnik V (*50*), both vaccines initially employing two doses, and Ad26 for Ad26.COV2.S which originated as a single dose vaccine (*48*). Sinopharm BBIBP-CorV (*51*) is an alum-adjuvanted, β-propiolactone-inactivated SARS-CoV-2 viral vaccine which initially utilized a two dose regimen.

Here, we set out to evaluate the influence of the SARS-CoV-2 S glycoprotein conformation on plasma neutralizing activity, which is a correlate of protection against COVID-19. To understand the molecular basis of elicitation of neutralizing antibodies elicited by a wide range of COVID-19 vaccines in humans and how to modulate their magnitude and breadth, we assessed the specificity of S-directed antibody responses, the relationship between antibody binding titers and neutralization potency, and the relative contribution of the RBD and the NTD to vaccine-mismatched cross-neutralizing activity against SARS-CoV-2 variants.

## RESULTS

### Prefusion SARS-CoV-2 S stabilization reduces the fraction of antibodies recognizing an off-target conformational state

**Fig. 1.**
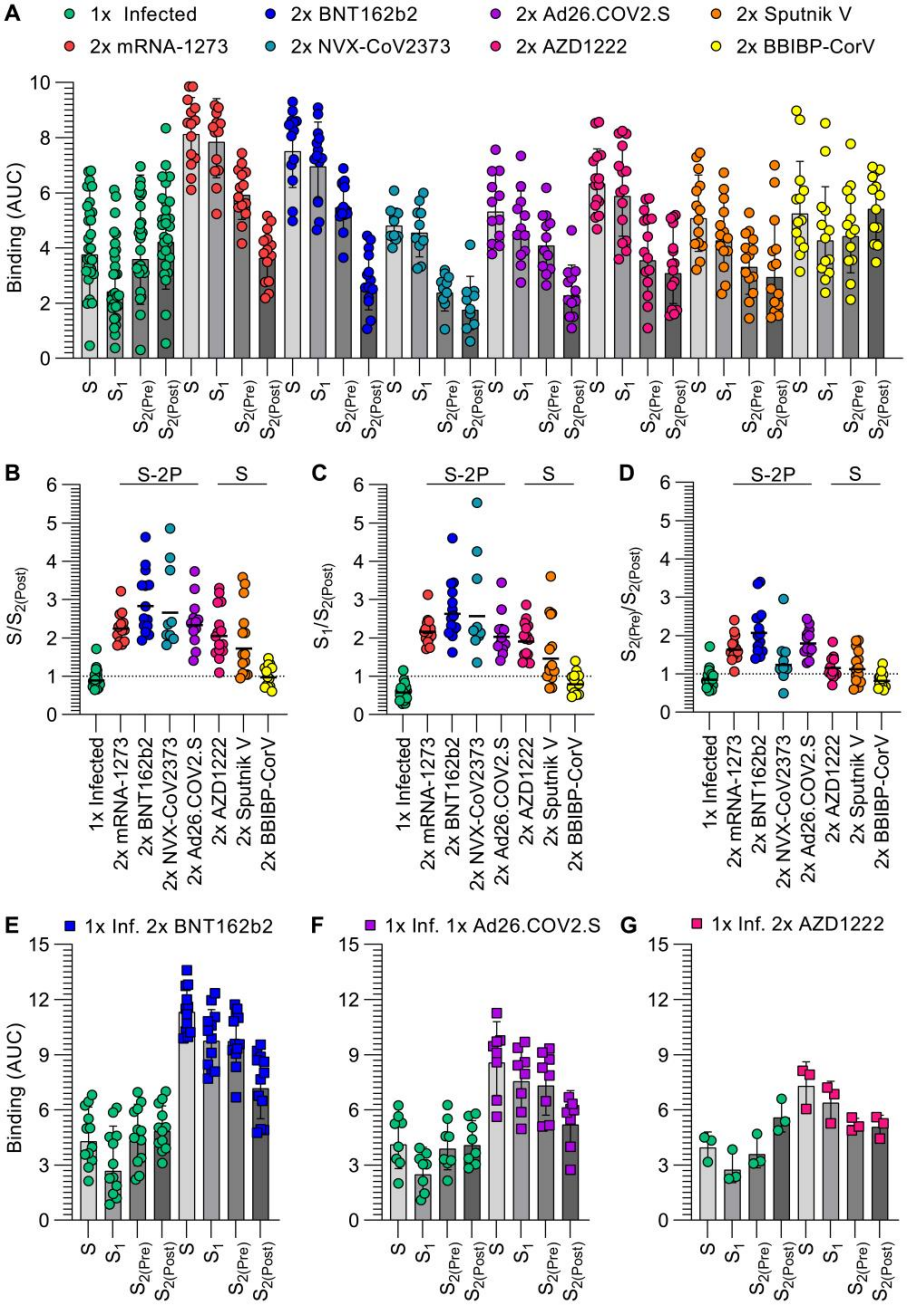
Prefusion SARS-CoV-2 S stabilization reduces the fraction of antibodies recognizing off-target conformational states. (**A**) IgG binding titers elicited by SARS-CoV-2 infection or vaccination against prefusion S (S), the S_1_ subunit, and the S_2_ subunit in the prefusion (S_2(Pre)_) and postfusion (S_2(Post)_) conformations, as measured by ELISA. Statistical analyses are shown in Tables S3-S4. (**B-D**) Ratio of plasma IgG binding titers against prefusion S (B), the S_1_ subunit (C), and the S_2_ subunit in the prefusion conformation (S_2(Pre)_) (D) over the plasma IgG binding titers against the S_2_ subunit in the postfusion conformation (S_2(Post)_). Cohorts labeled “S-2P” received vaccines encoding for or containing 2P prefusion-stabilizing S mutations whereas cohorts labeled “S” received vaccines lacking those mutations. Statistical analysis are shown in Tables S5 (**E-G**) IgG binding titers before and after vaccination with two doses of BNT162b2 (E), one dose of Ad26.COV2.S (F), or two doses of AZD1222 (G) in longitudinal cohorts of individuals previously infected with SARS-CoV-2. Statistical analyses are shown in Table S6 and Table S7. 1x infected samples (n = 28) were obtained 26–78 days (mean 42) post symptom onset, 2x mRNA-1273 samples (n = 14) were obtained 6–50 days (mean 15) post second dose, 2x BNT162b2 samples (n = 14) were obtained 6–33 days (mean 14) post second dose, 2x NVX-CoV2373 samples (n = 10) were obtained 17–168 days (mean 93–119) post second dose, 2x Ad26.COV2.S samples (n = 12) were obtained 12–16 days (mean 14) post second dose, 2x AZD1222 samples (n = 15) were obtained ~30 days post second dose, 2x Sputnik V samples (n = 14) were obtained 60–90 days post second dose, BBIBP-CorV samples (n = 13) were obtained 15–102 days (mean 71) post second dose, 1x Infected 2x BNT162b2 samples (n = 12) were obtained 10–32 days (mean 16) post second dose, 1x Infected 1x Ad26.COV2.S samples (n = 8) were obtained 12–112 days (mean 38) post first dose, and 1x Infected 2x AZD1222 samples (n = 3) were obtained ~30 days post second dose. Each point represents a single patient plasma sample from one representative out of at least two independent experiments consisting of different antigens, shaded bars represent the geometric mean, and error bars represent the geometric standard deviation. AUC was determined after log transforming the plasma dilution and these data are shown in Figures S5 and S6. Patient demographics are shown in Table S2.

To understand the specificity of S-directed antibody responses elicited by vaccination or infection, we evaluated plasma IgG binding titers against thoroughly validated prefusion-stabilized SARS-CoV-2 S trimer, the S_1_ subunit, the NTD, the RBD, and the S_2_ subunit (fusion machinery) in the prefusion (S_2(Pre)_) and postfusion (S_2(Post)_) states (**Fig S1-S3 and Table S1**). We determined a cryo–electron microscopy structure of S_2(Pre)_ which is presented in more detail in **Fig S2-S3 and Table S1**. Our panel includes samples from individuals who were not previously exposed to SARS-CoV-2 and received two doses of Moderna mRNA-1273, Pfizer/BioNTech BNT162b2, Novavax NVX-CoV2373, Janssen Ad26.COV2.S, AstraZeneca AZD1222, Gamaleya Research Institute Sputnik V, or Sinopharm BBIBP-CorV. We benchmarked these samples against COVID-19 human convalescent plasma obtained before January 2021, likely resulting from exposure to a Wuhan-Hu-1-related SARS-CoV-2 strain based on the date of symptom onset (**Table S2**) (*52*). Individuals that received two doses of mRNA-1273 or BNT162b2 had the highest prefusion S binding titers (geometric mean titers (GMTs) 8.1 and 7.5, respectively) whereas infected individuals had the lowest and most heterogeneous prefusion S binding titers (GMT 3.8), as assessed by enzyme-linked immunosorbent assays (ELISAs). Individuals that received two doses of NVX-CoV2373, Ad26.COV2.S, AZD1222, Sputnik V, and BBIBP-CorV had intermediate prefusion S binding GMTs (4.8, 5.3, 6.3, 5.0, and 5.2, respectively), although samples of individuals vaccinated with NVX-CoV2373 were collected ~2–3 months later than the other cohorts relative to the second vaccine dose (*53*, *54*). A similar trend and cohort grouping from highest (mRNA-1273 and BNT162b2) to intermediate (NVX-CoV2373, Ad26.COV2.S, AZD1222, Sputnik V, and BBIBP-CorV) and lowest (infected) binding titers, were observed when using the S_1_ subunit, the NTD, or the RBD as ELISA antigens (**Fig 1A****, Fig S4-S5, Table S3**). Vaccination with two doses of mRNA-1273, BNT162b2, NVX-COV2373, Ad26.COV2.S, AZD1222, and Sputnik V resulted in greater binding titers against S_1_ compared to S_2(Pre)_ and S_2(Pre)_ compared to S_2(Post)_, whereas infection or two doses of BBIBP-CorV resulted in greater binding titers against S_2(Post)_ compared to S_2(Pre)_ and S_2(Pre)_ compared to S_1_ **(****Fig 1A****, Fig S5, Table S4)**.

To assess the impact of prefusion S stabilization on vaccine-elicited antibody responses, we compared prefusion S, S_1_, and S_2(Pre)_-directed antibody titers to postfusion S_2_-directed antibodies across all seven vaccines and infection. Vaccination with two doses of mRNA-1273, BNT162b2, NVX-CoV2373, or Ad26.COV2.S elicited polyclonal plasma antibodies with higher S/S_2(Post)_ binding ratios (2.2, 2.8, 2.7, and 2.3, respectively) than two doses of AZD1222 and Sputnik V vaccines (2.1 and 1.7, respectively). Infection or two dose BBIBP-CorV vaccination elicited the lowest S/S_2(Post)_ ratios (0.9 and 1.0, respectively) **(****Fig 1B****, Table S5)**. This indicates preferential targeting of prefusion S by antibodies elicited by most vaccines, and particularly those that contain the ‘2P’ prefusion-stabilizing S mutations. Infection resulted in a S_1_/S_2(Post)_ binding ratio of 0.6 whereas two-dose vaccination with mRNA-1273, BNT162b2, NVX-CoV2373, Ad26.COV2.S, AZD1222, Sputnik V, and BBIBP-CorV resulted in S_1_/S_2(Post)_ binding ratios of 2.2, 2.6, 2.6, 2.0, 1.9, 1.5, and 0.8, respectively, thereby following the same trend as S/S_2(Post)_ binding ratios (**Fig 1C****, Table S5**). Vaccines containing prefusion-stabilizing mutations therefore elicited a higher proportion of S- and S_1_ relative to S_2(Post)_-directed polyclonal plasma antibodies compared to vaccines lacking such mutations or infection. Infection resulted in a S_2(Pre)_/S_2(Post)_ binding ratio of 0.9 whereas two dose vaccination with mRNA-1273, BNT162b2, NVX-CoV2373, Ad26.COV2.S, AZD1222, Sputnik V, and BBIBP-CorV elicited S_2(Pre)_/S_2(Post)_ binding ratios of 1.6, 2.1, 1.2, 1.8, 1.2, 1.1, and 0.8, respectively (**Fig 1D****, Table S5**). Prefusion-stabilized vaccines therefore elicited comparable or greater prefusion S_2_ over postfusion S_2_-directed antibody responses, relative to other vaccines, with the exception of NVX-CoV2373 which was characterized by low S_2(Pre)_ over S_2(Post)_ antibody titers, possibly due to the vaccine formulation or later timing of blood draw post second dose. Collectively, these data point to reduced elicitation of S_1_-directed relative to postfusion S_2_-directed antibodies in infected subjects or two dose BBIBP-CorV vaccinees. This is likely due to S_1_ shedding and S_2_ refolding to the postfusion conformation at the surface of authentic virions or infected cells (*55*–*57*), or as a result of the β-propiolactone inactivation procedure utilized by Sinopharm (*58*).

Previous studies demonstrated that COVID-19 vaccination of individuals previously infected with SARS-CoV-2 elicits high antibody binding and neutralizing titers (*59*–*62*). We therefore set out to assess and compare how antibody binding responses are affected upon vaccination of previously infected subjects with two doses of BNT162b2, one dose of Ad26.COV2.S, or two doses of AZD1222, corresponding to primary vaccine series dosing schemes. Although vaccination markedly enhanced the magnitude of antibody binding responses against all antigens tested, different vaccines led to distinct magnitudes of boosting. Post-vaccination to pre-vaccination prefusion S binding titers increased 2.6 times after two doses of BNT162b2, 2.1 times after a single dose of Ad26.COV2.S, and 1.8 times after two doses of AZD1222 **(****Fig 1E-G****, Fig S6, Table S6, Table S7)**. Whereas we observed S/S_2(Post)_ binding ratios comprised between 0.7–1.0 before vaccination, they rose to 1.6 for BNT162b2 and Ad26.COV2.S and 1.4 for AZD1222 after vaccination **(****Fig 1E-G****, Fig S6, Table S6, Table S7)**. Due to the metastable nature of the S trimer which is prone to shedding the S_1_ subunit and refolding to form postfusion trimers (*11*, *44*, *55*, *57*, *63*), the absence of prefusion-stabilizing S mutations in the AZD1222 vaccine might explain the slightly lower S/S_2(Post)_ binding ratios relative to BNT162b2 and Ad26.COV2.S. These data show that immunization with any of these three vaccines after infection skewed antibody responses preferentially toward prefusion S relative to postfusion S_2_, unlike infection only.

### SARS-CoV-2 neutralization is determined by S_1_ subunit targeting antibodies

**Fig. 2.**
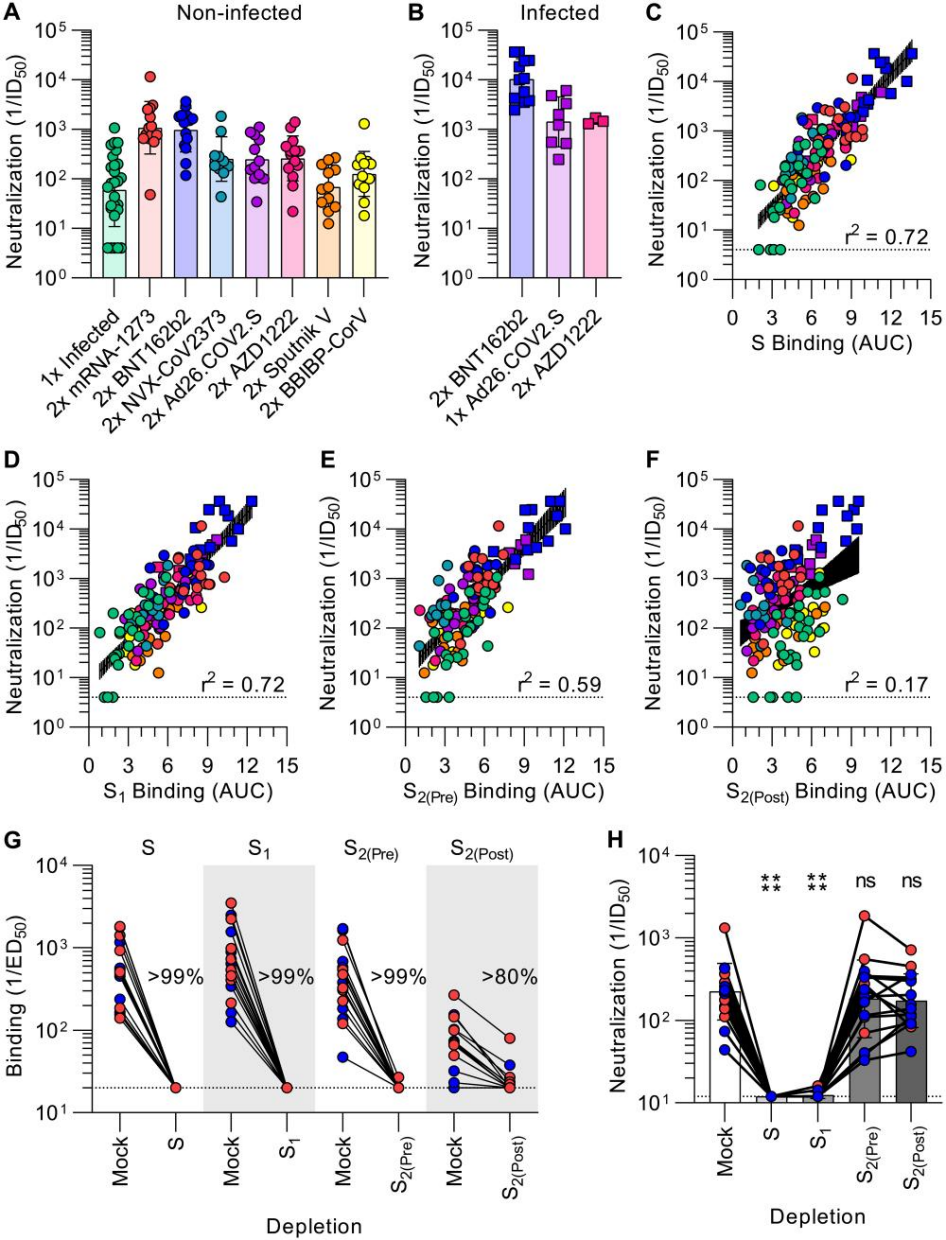
SARS-CoV-2 neutralization is determined by S_1_ subunit targeting antibodies. **A-B**, SARS-CoV-2 S pseudotyped VSV neutralization titers elicited by infection or vaccination (A), or vaccination following infection (B). The dotted line is the limit of detection, the colored bars are GMTs and the black error bars are geometric standard deviations. Colored points are the neutralizing geometric means of subjects after 2–4 experimental repeats consisting of different batches of pseudovirus, representative normalized curves are shown in Fig S7- S8, and statistical analyses are shown in Tables S7-S8. (**C-F**) Correlation between plasma neutralizing activity and prefusion S (C), S_1_, (D), prefusion S_2_ (E), and postfusion S_2_ (F) binding titers shown with a linear regression fit to the log of neutralization titers. The black shaded regions represent 95% confidence intervals. P < 0.0001 for all four panels. (**G-H**) Binding (G) and neutralization (H) titers resulting from depletion of polyclonal plasma antibodies targeting S, S_1_, prefusion S_2_, and postfusion S_2_. Each point is a patient plasma sample from one representative out of two independent experiments consisting of different batches of antigen and pseudovirus, shaded bars represent the geometric mean, and error bars represent the geometric standard deviation. Red points correspond to individuals vaccinated with two doses of mRNA-1273 whereas blue points correspond to individuals vaccinated with two doses of BNT162b2. Statistical significance between groups of data, relative to mock depletion, were determined by ratio paired Wilcoxon rank test and ns > 0.05, ****P < 0.0001. Mock consists of depletion carried out with beads lacking immobilized antigen. Binding data are shown in Fig S10 and dose-response neutralization curves are shown in Fig S11. Patient demographics are shown in Table S2.

To investigate the relationship between antibody binding titers and neutralization potency, we determined half-maximum inhibitory dilutions of the aforementioned plasma samples using a vesicular stomatitis virus (VSV) pseudotyped with the Wuhan-Hu-1 S glycoprotein harboring the D614G substitution (G614) and VeroE6 cells stably expressing TMPRSS2 (*64*). As a direct reflection of prefusion S and S_1_ binding titers, mRNA-1273 and BNT162b2 vaccinee plasma exhibited the highest neutralization potencies (GMTs 1080 and 968, respectively) whereas the neutralizing activity of previously infected individuals was the weakest among all groups (GMT 60) **(****Fig 2A****, Fig S7A-C, Table S8)**. Infection elicited the most heterogeneous humoral immune responses as defined by the wide spread of prefusion S binding and associated neutralizing antibody titers compared to other groups **(****Fig 1A** **and** **Fig 2A****)**. Subjects vaccinated twice with NVX-CoV2373, Ad26.COV2.S, AZD1222, Sputnik V, and BBIBP-CorV had neutralizing titers of 252, 247, 259, 69, and 126, respectively **(****Fig 2A****, Fig S7D-H, Table S8)**, although we note that NVX-CoV2373 samples were obtained the furthest from peak titers due to the design of the clinical trial from which they were obtained (*53*, *54*) **(Table S2)**. Individuals previously exposed to SARS-CoV-2 reached neutralizing GMTs of 10232 after two doses of BNT162b2 and 1479 after two doses of AZD1222 **(****Fig 2B****, Fig S8A, Table S9)**, corresponding to respective increases of 10.5-fold and 5.7-fold over those that had not been previously exposed to SARS-CoV-2 (*59*–*62*). Plasma from subjects previously infected with SARS-CoV-2 reached a neutralizing GMT of 1421 after a single dose of Ad26.COV2.S **(****Fig 2B****, Fig S8, Table S9)**, a 5.8-fold enhancement relative to those who received two doses of Ad26.COV2.S. Thus, vaccination of previously infected individuals elicits neutralizing antibody titers greater than administration of two doses of mRNA-1273 or BNT162b2 in naive individuals, in line with previous reports (*59*–*62*).

We observed a strong positive correlation between in vitro plasma inhibitory activity and the magnitude of antibody responses against the prefusion-stabilized S trimer for all vaccines evaluated and for infection-elicited polyclonal antibodies **(****Fig 2C****)**. Furthermore, we observed a comparable positive correlation between neutralizing activity and S_1_ binding antibody responses, suggesting a key role of S_1_-directed antibodies for SARS-CoV-2 neutralization (**Fig 2D**). Neutralizing antibody titers were also positively correlated with NTD- and RBD-specific binding titers **(Fig S9)**, in line with these two domains being part of the S_1_ subunit as well as the main targets of neutralizing antibodies upon infection or vaccination (*17*, *25*, *59*, *65*–*67*). The rapid accumulation of amino acid residue mutations in the SARS-CoV-2 S_1_ subunit throughout the COVID-19 pandemic (*68*) might therefore reflect, at least in part, the selective pressure exerted by host neutralizing antibodies. Although prefusion S_2_ antibody binding titers positively correlated with neutralization potency **(****Fig 2E****)**, postfusion S_2_ responses did not **(****Fig 2F****)**, indicating that antibodies targeting postfusion S are likely weak or not able to block viral entry. These results underscore the benefits of eliciting antibody responses targeting the prefusion S conformation, and particularly the S_1_ subunit, to maximize plasma neutralizing activity, and the higher quality of humoral immune responses elicited by vaccination with most platforms compared to natural infection.

To obtain a quantitative understanding of the relationship between S conformation and plasma neutralizing activity, we depleted polyclonal antibodies using prefusion S, the S_1_ subunit, prefusion S_2_, or postfusion S_2_ from the plasma of vaccinees who received two doses of mRNA-1273 or of BNT162b2. Binding titers against the respective antigens were reduced by >99% for S, S_1_, and S_2(Pre)_ and > 80% for S_2(Post)_ as determined by ELISA, confirming effective antigen-specific antibody removal **(****Fig 2G****, Fig S10)**. Depletion of prefusion S or S_1_ subunit-targeting antibodies resulted in a near-complete loss of neutralizing activity whereas depletion using prefusion or postfusion S_2_ had no detectable impact **(****Fig 2H****, Fig S11)**. These results clearly demonstrate that virtually all plasma neutralizing activity targets prefusion S, which is reminiscent of findings made for the respiratory syncytial virus fusion (F) glycoprotein (*69*, *70*). The coronavirus S glycoprotein, however, mediates both receptor binding and membrane fusion whereas respiratory syncytial virus F solely promotes membrane fusion. Furthermore, our data show that antibodies targeting the S_1_ subunit, and not the S_2_ subunit, account for vaccine-elicited plasma neutralizing activity. It is therefore S_1_ subunit shedding rather than S_2_ conformational changes associated with the prefusion to postfusion transition that leads to a marked loss of potency due to the fact that most neutralizing antibodies target the RBD (*17*, *65*, *71*) and the NTD (*25*, *26*, *28*).

### SARS-CoV-2 variant cross-neutralization is determined by RBD-specific antibodies

**Fig. 3.**
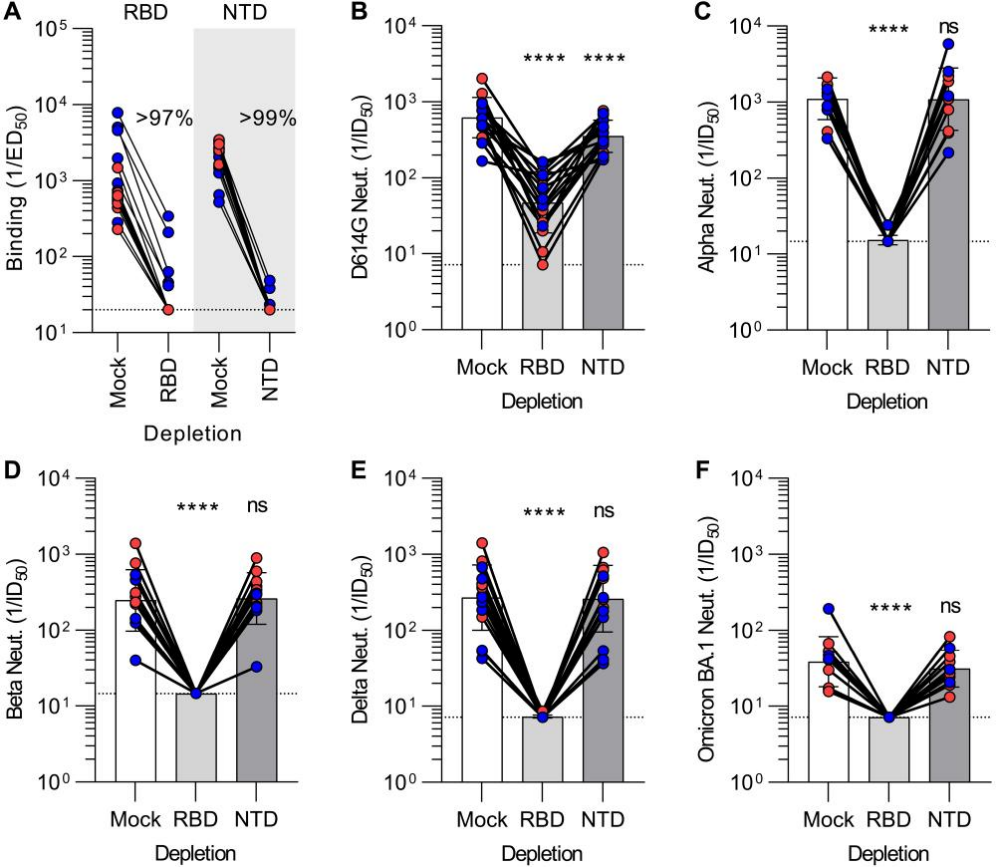
Vaccine-elicited broad neutralization of SARS-CoV-2 variants is mediated by RBD-directed antibodies. (**A**) Plasma IgG binding titers resulting from mock, Wuhan-Hu-1 RBD (left) and Wuhan-Hu-1 NTD (right) depletion of polyclonal antibodies. (**B-F**) Plasma neutralizing activity against G614 S VSV (B), Alpha S VSV (C), Beta S VSV (D), Delta S VSV (E) and Omicron BA.1 S VSV (F) after mock, Wuhan-Hu-1 RBD, or Wuhan-Hu-1 NTD depletion of polyclonal antibodies. We note that mock depleted BA.1 S VSV neutralization is dampened relative to G614 S VSV, in agreement with previous findings (*53*, *84*, *85*). Each point corresponds to a single patient plasma sample from one representative out of two independent experiments consisting of different batches of antigen and pseudovirus, shaded bars represent the geometric mean, and error bars represent the geometric standard deviation. Red points correspond to individuals vaccinated with two doses of mRNA-1273 whereas blue points correspond to individuals vaccinated with two doses of BNT162b2. Mock consists of depletion carried out with beads lacking immobilized antigen. Statistical significance between groups of data, relative to mock depletion, were determined by ratio paired Wilcoxon rank test and ns > 0.05, ****P < 0.0001. Fit binding curves are shown in Fig S12 and dose-response neutralization curves are shown in Fig S13. Patient demographics are shown in Table S2.

To evaluate the relative contribution of the RBD and the NTD to cross-neutralizing activity against SARS-CoV-2 variants, we depleted mRNA vaccinee plasma samples of antibodies recognizing each of these two antigens **(****Fig 3A****, Fig S12)**. Plasma neutralizing activity was subsequently determined against VSV pseudotyped with the G614 S glycoprotein, Alpha (B.1.1.7) S, Beta (B.1.351) S, Delta (B.1.617.2) S, or Omicron BA.1 (B.1.1.529) S using VeroE6 cells stably expressing TMPRSS2 (*64*, *67*, *72*, *73*). G614 S VSV neutralization was significantly reduced upon depletion of RBD-directed antibodies and to a lesser extent after depletion of NTD-directed antibodies for all samples tested (**Fig 3B****, Fig S13A**). This suggests that both RBD and NTD-targeting polyclonal plasma antibodies contribute to vaccine-elicited neutralizing activity of vaccine-matched Wuhan-Hu-1 SARS-CoV-2 pseudovirus (the D614G mutation has a very small effect on neutralization mediated by human plasma antibodies (*74*)). Strikingly, neutralization of the Alpha, Beta, Delta, and Omicron BA.1 S VSV pseudoviruses revealed a near-complete loss of neutralizing activity after depletion of RBD-directed plasma antibodies but no detectable contribution of NTD-directed antibodies **(****Fig 3C-F****, FigS13B-E)**. Since depletion of RBD-directed antibodies completely abrogated variant cross-neutralization, vaccine-elicited neutralization breadth is almost completely accounted for by antibodies targeting this domain. These data concur with (i) the marked antigenic variation of the SARS-CoV-2 NTD among variants and sarbecoviruses, which is associated with a narrow specificity of NTD neutralizing antibodies (*25*, *28*, *66*, *73*, *75*, *76*), and (ii) the description of multiple broadly neutralizing sarbecovirus antibodies recognizing distinct RBD antigenic sites (*14*–*16*, *18*, *77*–*83*).

## DISCUSSION

The discovery that most neutralizing activity in subjects infected with respiratory syncytial virus targets prefusion F led to subsequent stabilization of this conformational state through protein engineering and yielded clinically advanced vaccine candidates against this pathogen (*69*, *70*, *86*–*89*). We demonstrate here that prefusion SARS-CoV-2 S binding titers correlate with plasma neutralizing activity largely due to targeting of the S_1_ subunit, which comprises antigenic sites recognized by most neutralizing antibodies and is shed upon refolding. Targeting of the S_2_ subunit makes little contribution to vaccine-elicited polyclonal neutralizing activity due to the low frequency and weak potency of fusion machinery-directed neutralizing antibodies (*30*, *33*–*36*, *90*) although screening larger cohorts might help identifying subjects with a greater proportion of S_2_-targeting neutralizing antibodies. The data presented here in humans concur with mouse and non-human primate immunogenicity studies showing that prefusion-stabilized ‘2P’ S glycoproteins elicit greater neutralizing antibody titers than non-stabilized S trimers (*44*, *48*, *91*). These outcomes are likely resulting from the metastability of prefusion S and suggest that engineering next-generation S immunogens with additional prefusion-stabilizing mutations (e.g. ‘HexaPro S’ (*92*) or ‘VLFIP’ S (*93*)) could lead to vaccines eliciting even greater neutralizing antibody titers and resilience to SARS-CoV-2 variants. The identification of the RBD as the sole target of vaccine-elicited polyclonal antibodies broadly neutralizing SARS-CoV-2 variants is reminiscent of recent reports describing broadly neutralizing sarbecovirus monoclonal antibodies isolated from infected subjects (*14*–*16*, *18*, *20*, *77*–*79*, *94*) and the rapid accumulations of mutations in the NTD (*25*, *66*, *73*, *75*, *76*). Although cross-variant plasma neutralization is determined by RBD-directed antibodies, we note that Fc-mediated effector functions, including antibody-dependent phagocytosis, cellular cytotoxicity, and complement activation, can play key roles for in vivo protection in addition to direct viral neutralization (*95*–*100*). These findings motivate the clinical development of RBD-based vaccines against SARS-CoV-2 (*37*, *101*–*105*) and sarbecoviruses (*106*–*109*) for future pandemic preparedness.

## MATERIALS AND METHODS

### Study design

To study the influence of the SARS-CoV-2 S conformation on plasma neutralizing activity, we collected human plasma samples from subjects that had received a primary vaccine series of the Moderna/NIAID mRNA-1273, Pfizer/BioNTech BNT162b2, Novavax NVX-CoV2373, AstraZeneca/Oxford AZD1222, Gamaleya Research Institute Sputnik V, Janssen Ad26.COV2.S and Sinopharm BBIBP-CorV with or without administration of homologous or heterologous boosters and with or without prior SARS-CoV-2 exposure. To understand the molecular basis of elicitation of neutralizing antibodies, we assessed the specificity of S-directed antibody responses for various S constructs, the correlation between antibody binding titers and neutralization potency, and the relative contribution of the RBD and the NTD to vaccine-matched and vaccine-mismatched cross-neutralizing activity against SARS-CoV-2 variants.

### Cell lines

Cell lines used in this study were obtained from ThermoFisher Scientific (HEK293T and Expi293F) or were kindly gifted by Florian Lempp (Vero-TMPRSS2 cells (*64*)). None of the cell lines used were authenticated or tested for mycoplasma contamination.

### Sample donors

Convalescent plasma, mRNA-1273, BNT162b2, and previously infected BNT162b2 and Ad26.COV2.S samples were obtained from the HAARVI study approved by the University of Washington Human Subjects Division Institutional Review Board (STUDY00000959). Some mRNA-1273 samples were obtained from individuals enrolled in the UWARN: COVID-19 in WA study approved by the University of Washington Human Subjects Division Institutional Review Board (STUDY00010350). Samples from NVX-CoV2373 immunized individuals were collected in the San Diego region by the La Jolla Institute for Immunology (*54*). This work was approved by the institutional review board (IRB) of the La Jolla Institute (IRB#: VD-214). Ad26.COV2.S samples were obtained from and approved by the Infectious Diseases Clinical Research Consortium. AZD1222 samples were obtained from the PolImmune-COVID study conducted by INGM and IRCCS Ca′ Granda Ospedale Maggiore Policlinico of Milan, approved by INMI “Lazzaro Spallanzani” Ethics Committee (286_2021). Sputnik V samples were obtained from healthcare workers at the hospital de Clínicas “José de San Martín”, Buenos Aires, Argentina. BBIBP-CorV samples were obtained from Aga Khan University, Karachi, Pakistan. Demographic data for these individuals are summarized in Table S2.

### Plasmid construction

The SARS-CoV-2 S-6P is as previously described (Hsieh et al., 2020) and placed into CMVR with an octa-his tag. The SARS-CoV-2 N-terminal domain:SARS-CoV-2 NTD (residues 14–307) with a C-terminal 8XHis-tag was sub-cloned in pCMV as previously described (McCallum et al., 2020). The SARS-CoV-2-RBD-Avi construct was synthesized by GenScript into pcDNA3.1- with an N-terminal mu-phosphatase signal peptide and a C-terminal octa-histidine tag, flexible linker, and avi tag (GHHHHHHHHGGSSGLNDIFEAQKIEWHE). The boundaries of the construct are N-_328_RFPN_331_ and _528_KKST_531_-C (Walls et al., 2020a). SARS-CoV-2 S G614 (Lempp et al. 2021) has a mu-phosphatase signal peptide beginning at Q14, a mutated S1/S2 cleavage site (SGAR), ends at residue K1211 and is followed by a TEV cleavage site, fold-on trimerization motif, and an 8× His tag in the pCMV vector. SARS-CoV-2 S_1_ has a mu-phosphatase signal peptide, mutated furin cleavage site to SGAS, D614G mutation, Rpk9 mutations (Y365F/F392W/V395I), ends at S686 followed by a 16GS linker, I53-50A, and Cterminal his tag. SARS-CoV-2 S_2_ has a mu-phsophatase signal peptide and begins at _686689_ with stabilizing mutations from the vFlip construct (Y707C/T883C, A892P, A899P, A942P, and V967P) (Olmedillas et al. 2021). It also contains a F970C-G999C disulfide to further lock into the prefusion (Simmerling). The construct ends at residue Q1208 and is followed by a fold-on trimerization motif,TEV cleavage site, an 8× His tag and avi tag in the CMVR vector. SARS-CoV-2 G614 S (YP 009724390.1), Alpha (B.1.1.7), Beta (B.1.351) and Delta (B.1.617.2), S genes were all placed into the HDM vector with a 21 residue C-terminal deletion, as previously described (*76*, *106*, *110*). The plasmids encoding the SARS-CoV-2 Omicron BA.1 S variant were generated by overlapping PCR mutagenesis of the wildtype plasmid, pcDNA3.1(+)-spike-D19 (*111*).

### Protein expression and purification

SARS-CoV-2 S subunits and domains were produced in Expi293F Cells (ThermoFisher Scientific) grown in suspension using Expi293 Expression Medium (ThermoFisher Scientific) at 37°C in a humidified 8% CO2 incubator rotating at 130 rpm. Cells grown to a density of 3 million cells per mL were transfected using the ExpiFectamine 293 Transfection Kit (ThermoFisher Scientific) and cultivated for 3–5 days. Proteins were purified from clarified supernatants using a nickel HisTrap HP affinity column (Cytiva) and washed with ten column volumes of 20 mM imidazole, 25 mM sodium phosphate pH 8.0, and 300 mM NaCl before elution on a gradient to 500 mM imidazole. To produce SARS-CoV-2 S in the postfusion state, SARS-CoV-2 S D614G was incubated with 1:1 w/w S2X58-Fab (*16*) and 10 ug/mL trypsin for one hour at 37°C before size exclusion on a Superose 6 Increase column (Cytivia). Proteins were buffer exchanged into 20 mM sodium phosphate pH 8 and 100 mM NaCl and concentrated using centrifugal filters (Amicon Ultra) before being flash frozen.

### Antibody expression and purification

RAY-53-IgG (Huang et al. 2021) was produced in ExpiCHO Cells (ThermoFisher Scientific) grown in suspension using ExpiCHO Expression Medium (ThermoFisher Scientific) at 37°C in a humidified 8% CO2 incubator rotating at 130 rpm. Cells grown to a density of 6 million cells per mL were transfected using the ExpiFectamine CHO Transfection Kit (ThermoFisher Scientific) and cultivated for 6–8 days. Proteins were purified from clarified supernatants using a Protein A affinity column (Cytiva) and washed with ten column volumes of 20 mM sodium phosphate pH 8.0 before elution with 0.1 M citric acid pH 3 into 1 M Tris-HCl pH 9. Proteins were buffer exchanged into 20 mM sodium phosphate pH 8 and 100 mM NaCl and concentrated using centrifugal filters (Amicon Ultra) before being flash frozen.

### Enzyme-linked immunosorbent assay (ELISA)

30 μL of 3 ug/mL of SARS-CoV-2 S, NTD, RBD, S_1_ S_2(Pre)_, or S_2(Post)_ diluted in PBS were incubated on a 384-well Nunc Maxisorp plate (ThermoFisher 464718) for one hour at 37°C. Plates were slapped dry before addition of 80 μL blocker Casein in PBS (ThermoFisher) and incubation for one hour at 37°C. Plates were slapped dry and a 1:4 serial dilution of plasma in 30 μL TBST was added and incubated for one hour at 37°C. Plates were slapped dry and washed 4x with TBST using a BioTek plate washer followed by addition of Invitrogen anti-Human IgG (ThermoFisher A18817) and one hour incubation at 37°C. Plates were once again slapped dry and washed 4x with TBST before addition of room temperature TMB Microwell Peroxidase (Seracare 5120–0083). The reaction was quenched after 1–2 minutes with 1 N HCl and the A450 of each well was read using a BioTek plate reader. Prism (GraphPad) area under curve (AUC) was used to analyze data following log transformation of dilution series.

### Pseudotyped VSV production

SARS-CoV-2 G614, Alpha (B.1.1.7), Beta (B.1.351), Delta (B.1.617.2), and Omicron BA.1 pseudotypes were prepared similarly as previously described (*76*). Briefly, HEK-293 T cells seeded in poly-D-lysine coated 100 mm dishes at ~75% confluency were washed five times with Opti-MEM and transfected using 24 μg of the S glycoprotein plasmid with Lipofectamine 2000 (Life Technologies). After 5 h at 37°C, media supplemented with 20% FBS and 2% PenStrep was added. After 20 hours, cells were washed five times with DMEM and cells were transduced with VSVΔG-luc before a 2 h incubation at 37°C. Infected cells were then washed an additional five times with DMEM prior to adding media supplemented with anti-VSV-G antibody (I1-mouse hybridoma supernatant diluted 1:25, from CRL-2700, ATCC) to reduce parental background. After 18–24 h, the supernatant was harvested and clarified by low-speed centrifugation at 2,500 g for 10 min. The supernatant was then filtered (0.45 μm) and concentrated 10 times using a 30 kDa MWCO centrifugal concentrator (Amicon Ultra). The pseudotypes were then aliquoted and frozen at −80°C.

### Pseudotyped VSV neutralization assay

To evaluate neutralization of G614, Alpha (B.1.1.7), Beta (B.1.351), Delta (B.1.617.2), and Omicron BA.1 pseudotypes by plasma of vaccinees or previously infected individuals, Vero-TMPRSS2 cells in DMEM supplemented with 10% FBS, 1% PenStrep, and 8 ug/mL puromycin were seeded at 60–70% confluency into white clear-bottom 96 well plates (Corning) and incubated at 37°C. The following day, a half-area 96-well plate (Greiner) was prepared with eight 3-fold serial plasma dilutions. An equal volume of DMEM with 1:25 pseudovirus and 1:25 anti-VSV-G antibody (I1-mouse hybridoma supernatant from CRL-2700, ATCC) was then added to the half-area plate. The mixture was incubated at room temperature for 20–30 minutes. Media was removed from the cells and 40 μL from each well (containing plasma and pseudovirus) was transferred to the 96-well plate seeded with Vero-TMPRESS2 cells and incubated at 37°C for 2 h. After 2 h, an additional 40 μL of DMEM supplemented with 20% FBS and 2% PenStrep was added to the cells. After 16–20 h, 40 μL of One-Glo-EX substrate (Promega) was added to each well and incubated on a plate shaker in the dark for 5 min. Relative luciferase units were read using a Biotek plate reader. Relative luciferase units were plotted and normalized in Prism (GraphPad): 100% neutralization being cells lacking pseudovirus and 0% neutralizing being cells containing virus but lacking plasma. Prism (GraphPad) nonlinear regression with “[inhibitor] versus normalized response with a variable slope” was used to determine ID50 values from curve fits with 2–3 repeats. 2–4 biological replicates were carried out for each sample.

### Depletion of SARS-CoV-2 S-binding antibodies from polyclonal plasma

400 μL of vortexed Invitrogen His-Tag Dynabeads (ThermoFisher 10104D) were aliquoted into microcentrifuge tubes and incubated on an Invitrogen DynaMag-2 Magnet (ThermoFisher 12–321-D) for two minutes. The supernatant was discarded and beads were washed with 300 μL TBST. After a two-minute incubation on the magnet, the supernatant was discarded and 100 μg of his-tagged S, S_1_, S_2(Pre)_, S_2(Post)_, NTD, or RBD in 300 μL TBST was left to incubate with the beads for 10–20 min at room temperature. The magnet was used and supernatant discarded. The beads were spun down, put on the magnet, and excess liquid was removed before addition of 15–20 μL of plasma of interest. The plasma was left to incubate at room temperature with the beads for 20–30 min before being removed.

### Negative stain EM sample preparation and data collection

Protein samples were diluted to 0.01 mg/mL immediately prior to adsorption to glow-discharged carbon-coated copper grids for ~30 sec prior to a 2% uranyl formate staining. Micrographs were recorded using the Leginon software on a 120 kV FEI Tecnai G2 Spirit with a Gatan Ultrascan 4000 4 k x 4 k CCD camera at 67,000 nominal magnification. The defocus ranged from −1.0 to −2.0 μm and the pixel size was 1.6 Å.

### CryoEM sample preparation, data collection and data processing

Three microliters of the recombinantly expressed and purified prefusion SARS-CoV-2 S_2_ subunit were loaded onto freshly glow discharged R 2/2 UltrAuFoil grids (200 mesh) (*112*), prior to plunge freezing using a vitrobot MarkIV (ThermoFisher Scientific) with a blot force of 0 and 6.5 sec blot time at 100% humidity and 22°C.

Data were acquired using an FEI Titan Krios transmission electron microscope operated at 300 kV and equipped with a Gatan K3 direct detector and Gatan Quantum GIF energy filter, operated in zero-loss mode with a slit width of 20 eV. Automated data collection was carried out using Leginon (*113*) at a nominal magnification of 105,000x with a physical pixel size of 0.843 Å. The dose rate was adjusted to 15 counts/pixel/s, and each movie was acquired in super-resolution mode fractionated in 75 frames of 40 ms. 7,859 micrographs were collected with a defocus range comprised between −0.5 and − 2.5 μm. Movie frame alignment, estimation of the microscope contrast-transfer function parameters, particle picking, and extraction were carried out using Warp (*114*).

Two rounds of reference-free 2D classification were performed using CryoSPARC (*115*) to select well-defined particle images. These selected particles were subjected to two rounds of 3D classification with 50 iterations each (angular sampling 7.5° for 25 iterations and 1.8° with local search for 25 iterations), using an ab-initio map as initial model, in Relion (*116*). 3D refinements were carried out using non-uniform refinement along with per-particle defocus refinement in CryoSPARC (*117*). Selected particle images were subjected to the Bayesian polishing procedure (*118*) implemented in Relion 3.0 before performing another round of non-uniform refinement in cryoSPARC followed by per-particle defocus refinement and again non-uniform refinement. Local resolution estimation, filtering, and sharpening were carried out using CryoSPARC. Reported resolutions are based on the gold-standard Fourier shell correlation (FSC) of 0.143 criterion and Fourier shell correlation curves were corrected for the effects of soft masking by high-resolution noise substitution (*119*, *120*).

### Model building and refinement

UCSF Chimera (*121*) and Coot (*122*) were used to fit and rebuild an atomic model derived from PDB 6VXX (*1*) into the cryoEM map. The model was subsequently refined and relaxed with Rosetta using sharpened and unsharpened maps (*123*, *124*). Model validation and analysis used MolProbity (*125*), EMringer (*126*), Phenix (*127*) and Privateer (*128*). Figures were generated using UCSF ChimeraX (*129*).

### Statistical analysis

Statistical significance analysis of differences between binding titers of the same antigen or neutralization titers, following immunization with different vaccines, was determined using Turkey’s multiple comparisons test. Statistical significance analysis of differences between binding titers of different antigens, following immunization with the same vaccine, was determined using paired Turkey’s multiple comparisons test. Statistical significance analysis of differences between mock and depleted samples was determined using ratio paired Wilcoxon rank test.

## Supplementary Materials

### Other Supplementary Material for this manuscript includes the following:

Data S1

MDAR Reproducibility Checklist

## Notes

View/request a protocol for this paper from *Bio-protocol*.
