# Supplementary material for: SARS-CoV-2 spike conformation determines plasma neutralizing activity elicited by a wide panel of human vaccines
Source: Sci Immunol. 2022 Nov 10:eadf1421. doi: 10.1126/sciimmunol.adf1421 (PMC9765460; doi:10.1126/sciimmunol.adf1421)
Supplement: Supplementary file 1 — Figs. S1 to S13 Tables S1 to S9 [file sciimmunol.adf1421_sm.pdf]

Science Immunology
AAAS

Supplementary Materials for

SARS-CoV-2 spike conformation determines plasma neutralizing activity elicited by a wide panel of human vaccines

John E. Bowen *et al.*

Corresponding author: David Veesler, dveesler@uw.edu

Sci. Immunol. **7**, eadf1421 (2022)
DOI: 10.1126/sciimmunol.adf1421

The PDF file includes:

Figs. S1 to S13
Tables S1 to S9

Other Supplementary Material for this manuscript includes the following:

Data S1
MDAR Reproducibility Checklist

Supplemental Material

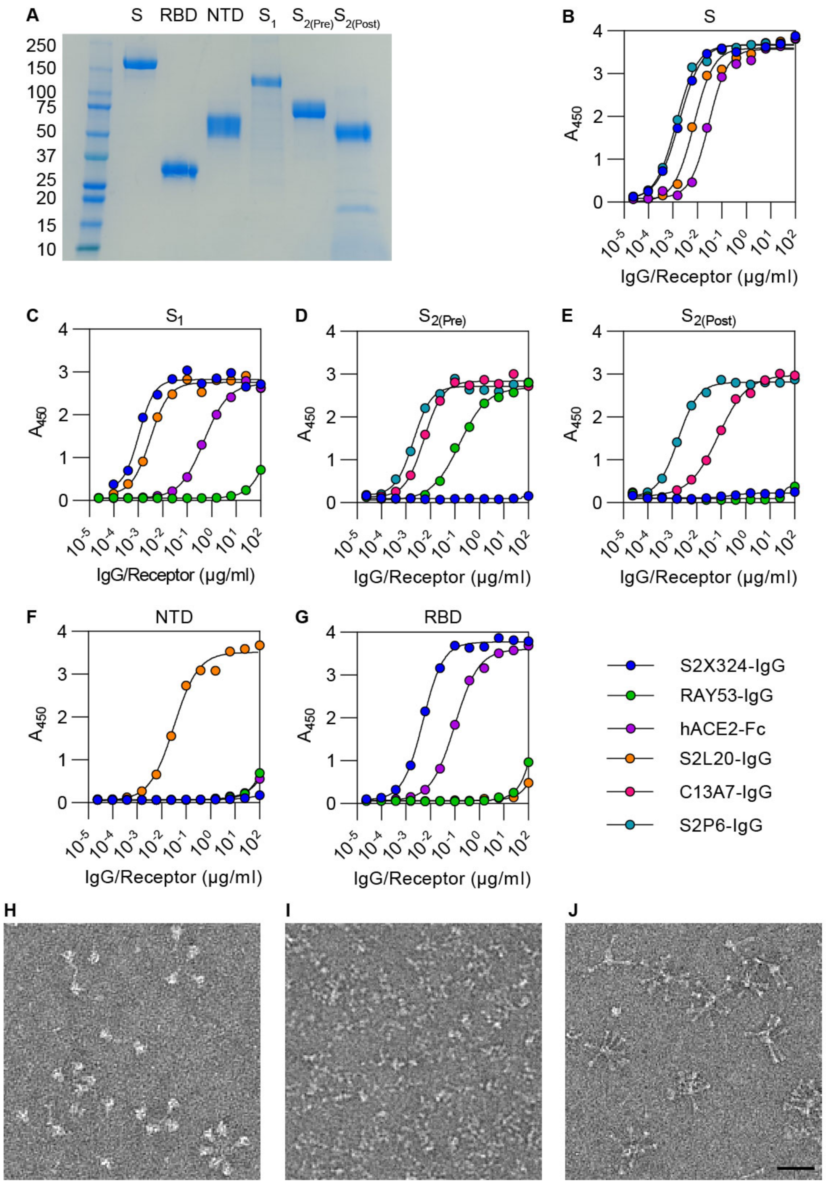

Figure S1. **Biophysical and antigenic characterization of the antigens used for binding and depletion experiments. A**, Reducing SDS-PAGE gel of SARS-CoV-2 S, the receptor-binding domain (RBD), the N-terminal domain (NTD), the S_1 subunit, and the S_2 subunit in the prefusion and postfusion conformations. **B-G**, Dose-response ELISA curves for binding of the RBD-targeting S2X324 IgG (*80*), the prefusion S_2-targeting RAY-53 IgG (*36*), the NTD-targeting S2L20 IgG (*25*), the S_2-targeting C13A7 IgG (*34*), the S_2 stem helix-targeting S2P6 IgG (*33*) and the human ACE2 ectodomain fused to an Fc fragment (hACE2-Fc) to S (B), S_1 (C), $S_{2(Pre)}$ (D) $S_{2(Post)}$ (E), NTD (F), and RBD (G). **H-J**, Representative negative stain electron micrograph of S (H), S_1 (I), and $S_{2(Post)}$ (J). Scale bar: 400 Å .

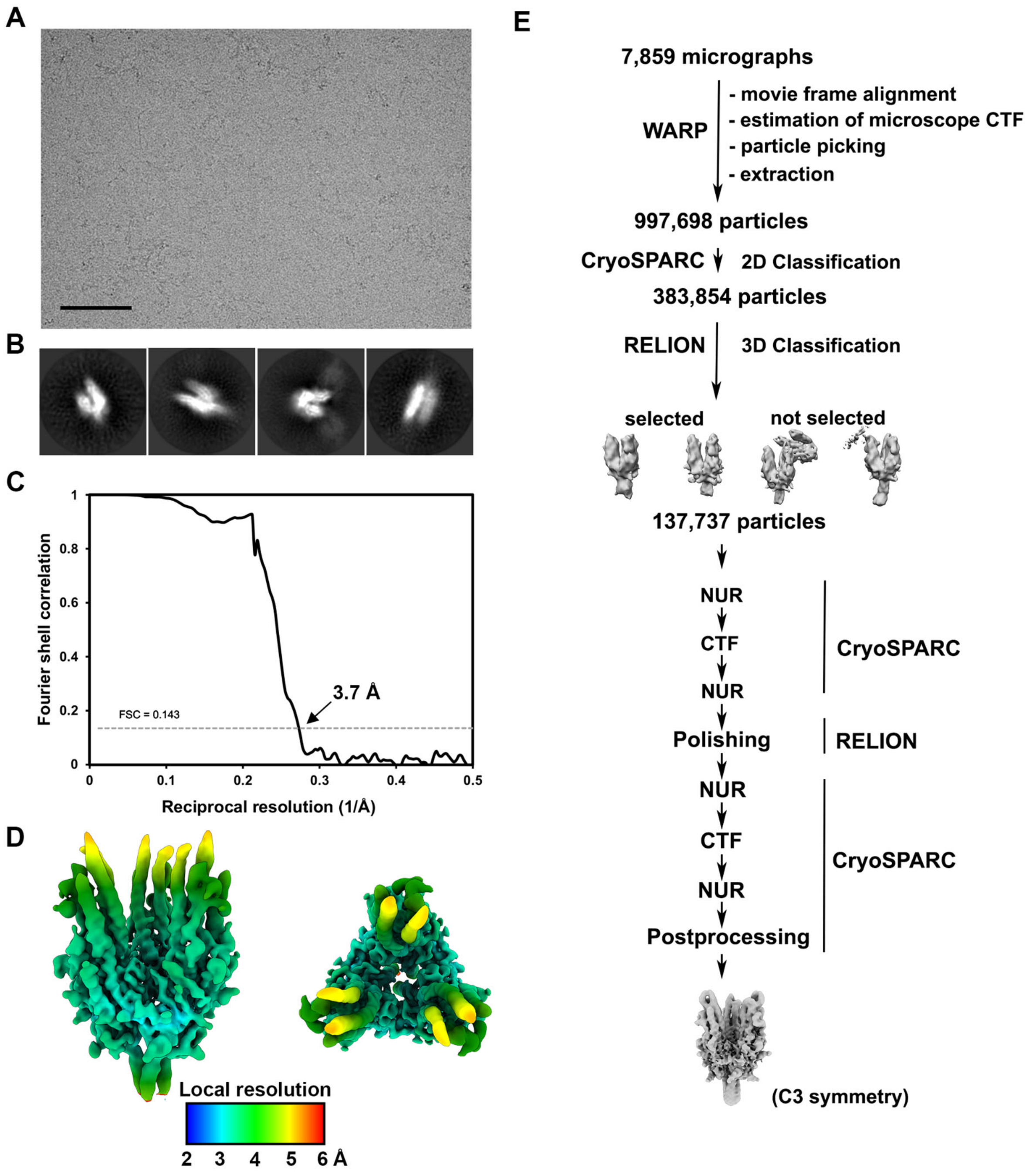

Figure S2. **CryoEM data processing of the prefusion SARS-CoV-2 S_2 subunit trimer dataset.** **A-B**, Representative electron micrograph (A) and 2D class averages (B) of SARS-CoV-2 S_2 embedded in vitreous ice. The scale bar represents 100 nm. **C**, Gold-standard Fourier shell correlation curve for the SARS-CoV-2 S_2 reconstruction. The 0.143 cutoff is indicated by a horizontal dashed line. **D**, Local resolution map calculated using CryoSPARC and plotted onto the unsharpened SARS-CoV-2 S_2 reconstruction viewed from the side (left, along the viral membrane) and the top (right, normal to the viral membrane). **E**, Data processing flowchart. CTF: contrast transfer function; NUR: non-uniform refinement.

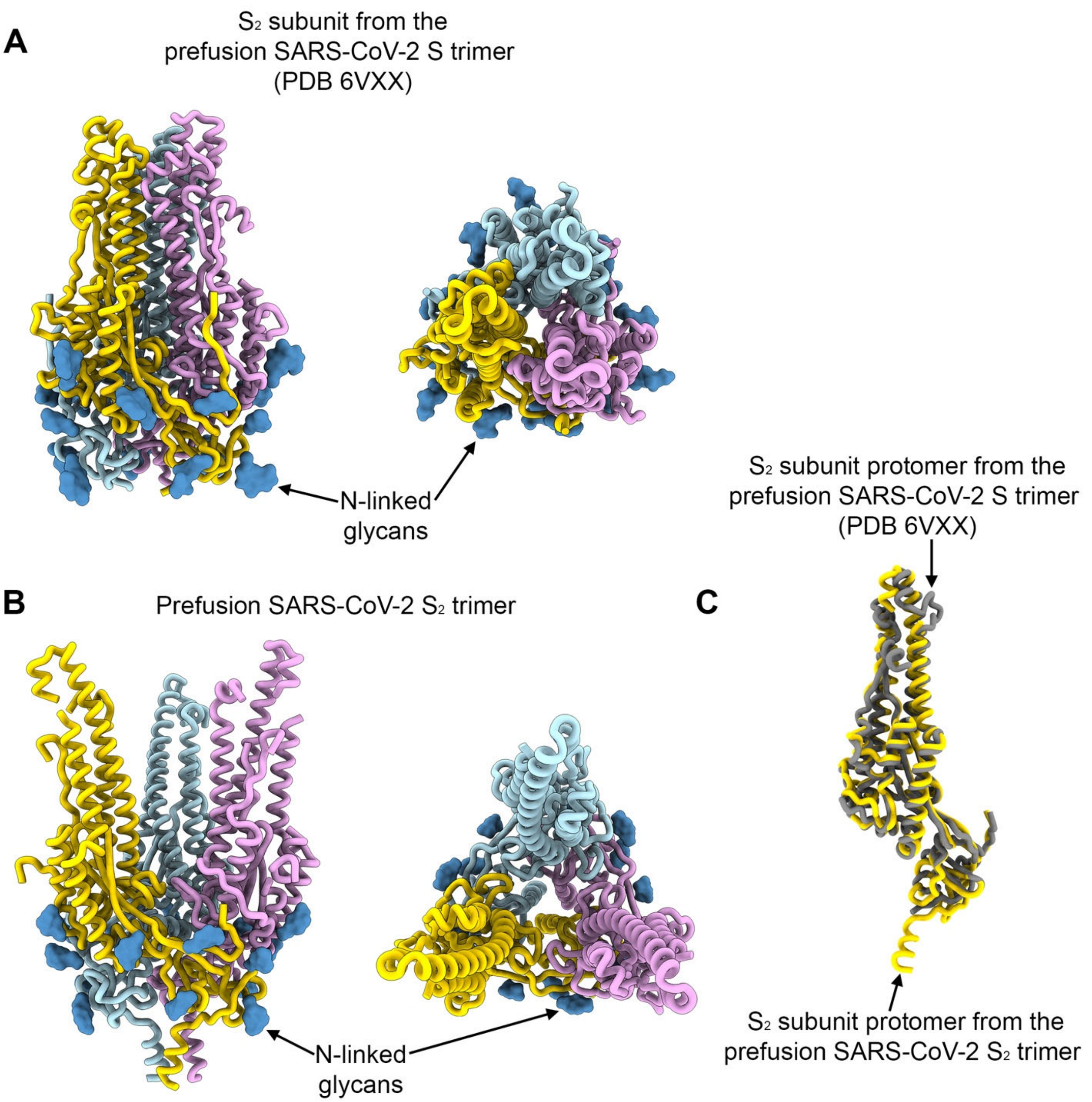

Figure S3. **Comparison of the fusion machinery architecture in prefusion SARS-CoV-2 S and S_2. A**, Ribbon diagram of the S_2 trimer present in the prefusion S ectodomain structure (PDB 6VXX) viewed along two orthogonal orientations. **B**, Ribbon diagram of the S_2 subunit ectodomain trimer structure viewed along two orthogonal orientations. **C**, Superimposition of one protomer from each of the two structures underscoring retention of the overall prefusion tertiary S_2 subunit structure (albeit with some deviation of the quaternary organization of the trimer).

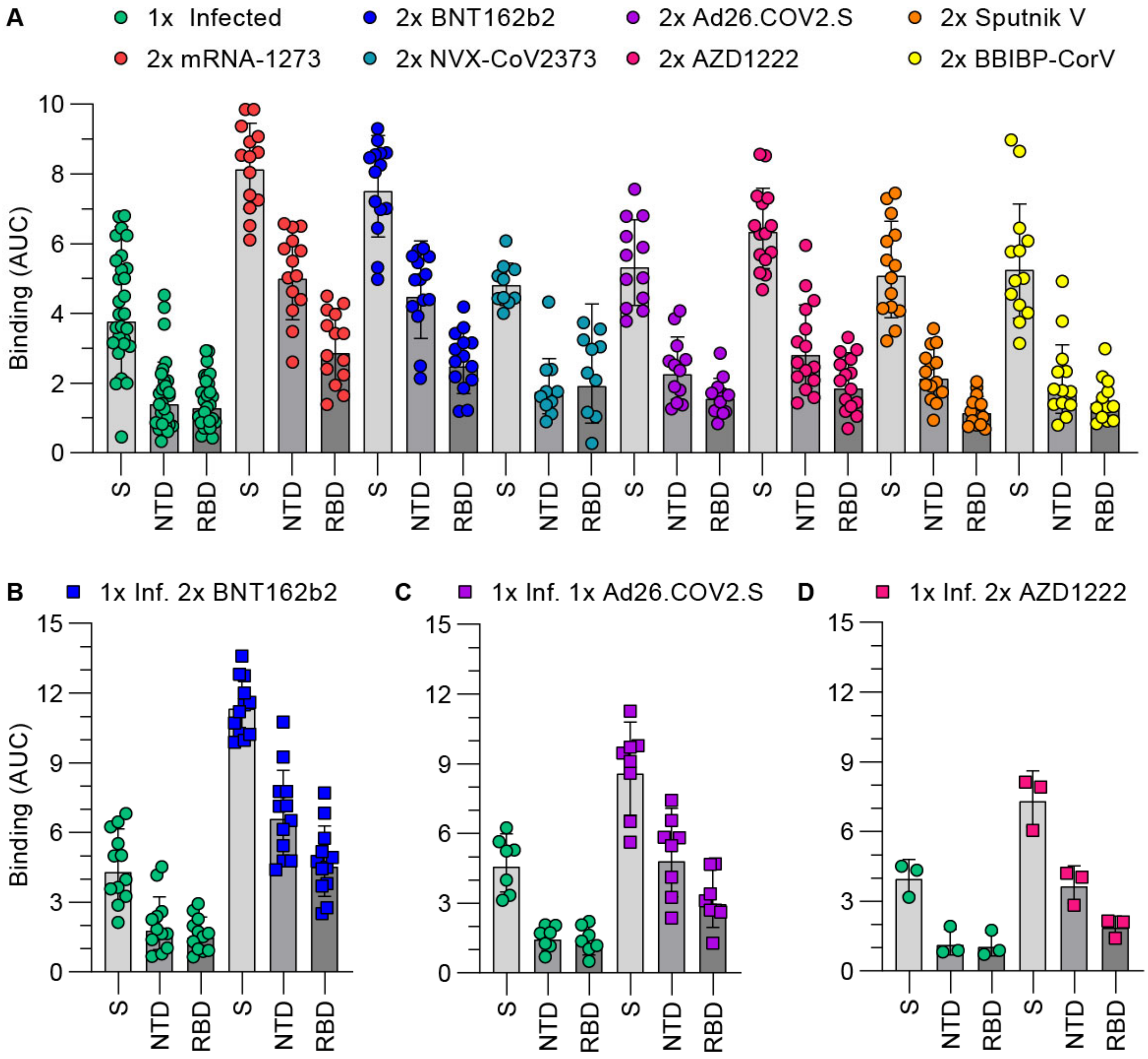

Figure S4. **A**, IgG binding titers elicited by SARS-CoV-2 infection or vaccination against the prefusion S (S), the N-terminal domain (NTD), and the receptor-binding domain (RBD), as measured by ELISA. Individuals were infected with a Wuhan-Hu-1-like strain or vaccinated with two doses of one of seven vaccines. **B-D**, IgG binding titers before and after vaccination with two doses of BNT162b2 (B), one dose of Ad26.COV2.S (C), or two doses of AZD1222 (D) in longitudinal cohorts of individuals previously infected with SARS-CoV-2. Each point consists of a representative result from a single patient plasma sample out of at least two experimental replicates consisting of different batches of each antigen. Bars represent geometric means and error bars represent geometric standard deviations. AUCs were determined after log transforming the plasma dilutions and the corresponding dose-response curves are shown in Fig S5-S6.

A 1x Infected

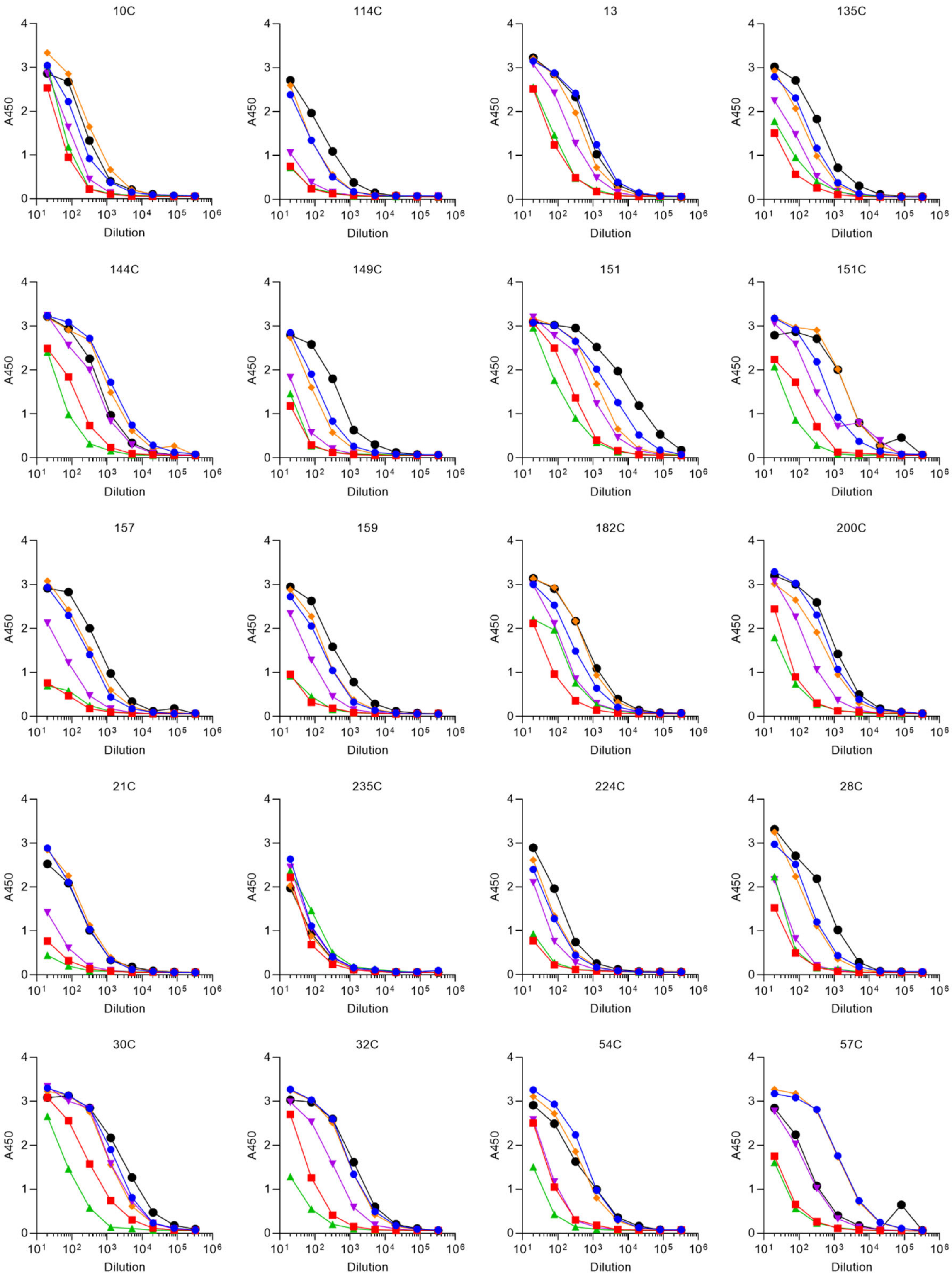

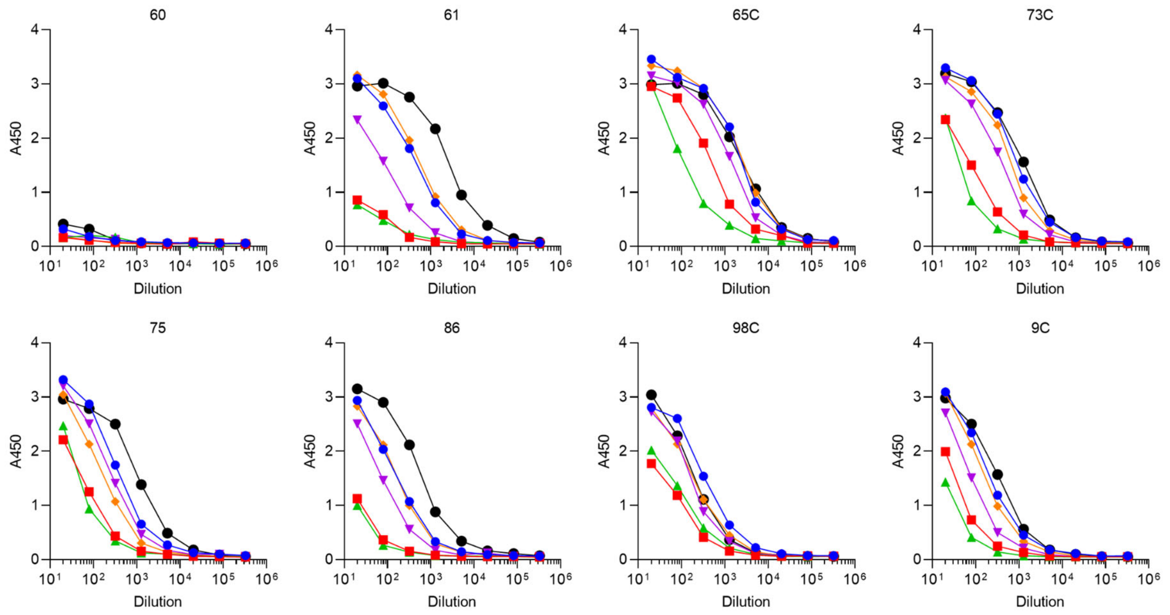
60
61
65C
73C
75
86
98C
9C
A450
Dilution

B 2x mRNA-1273

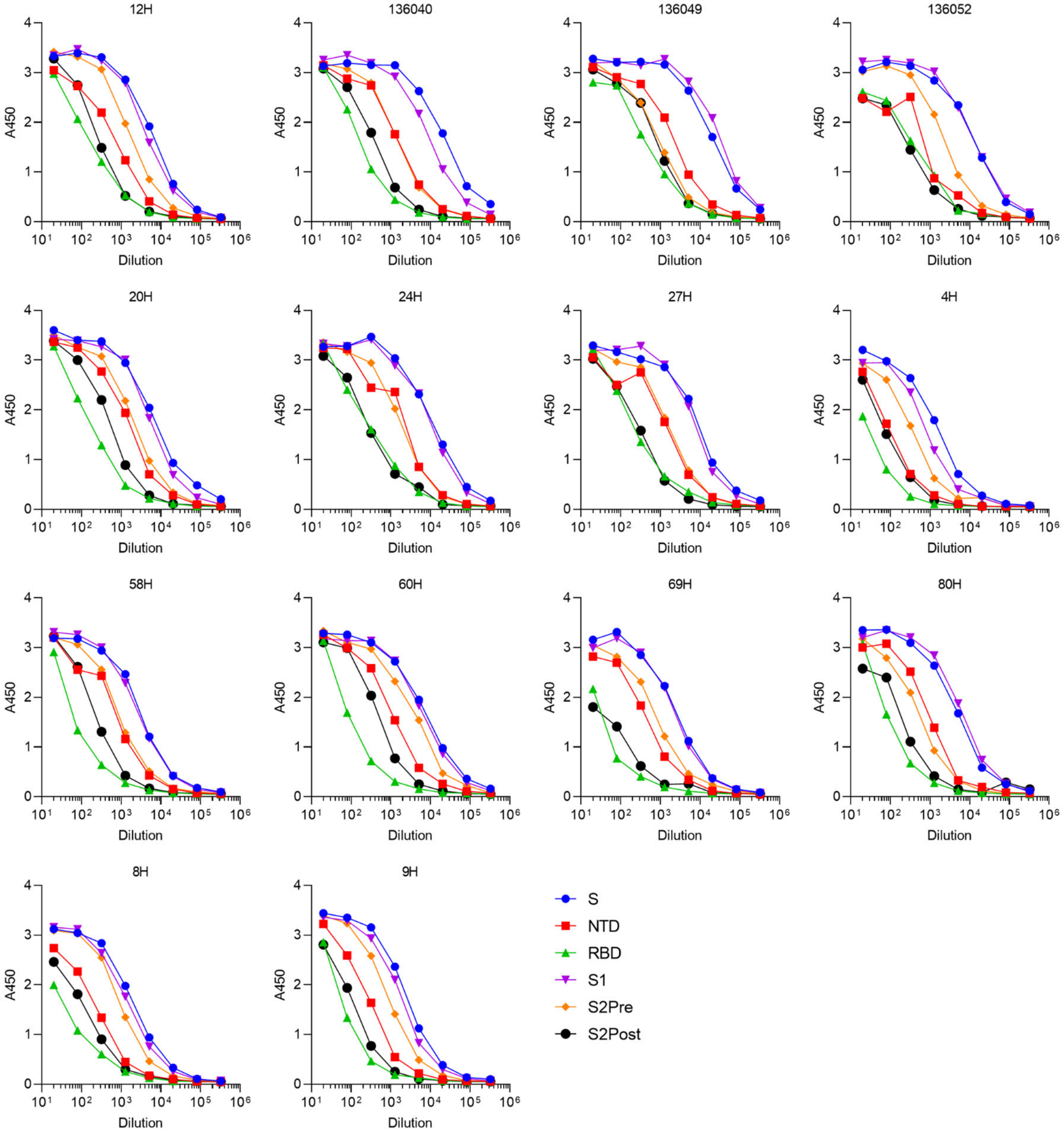

C 2x BNT162b2

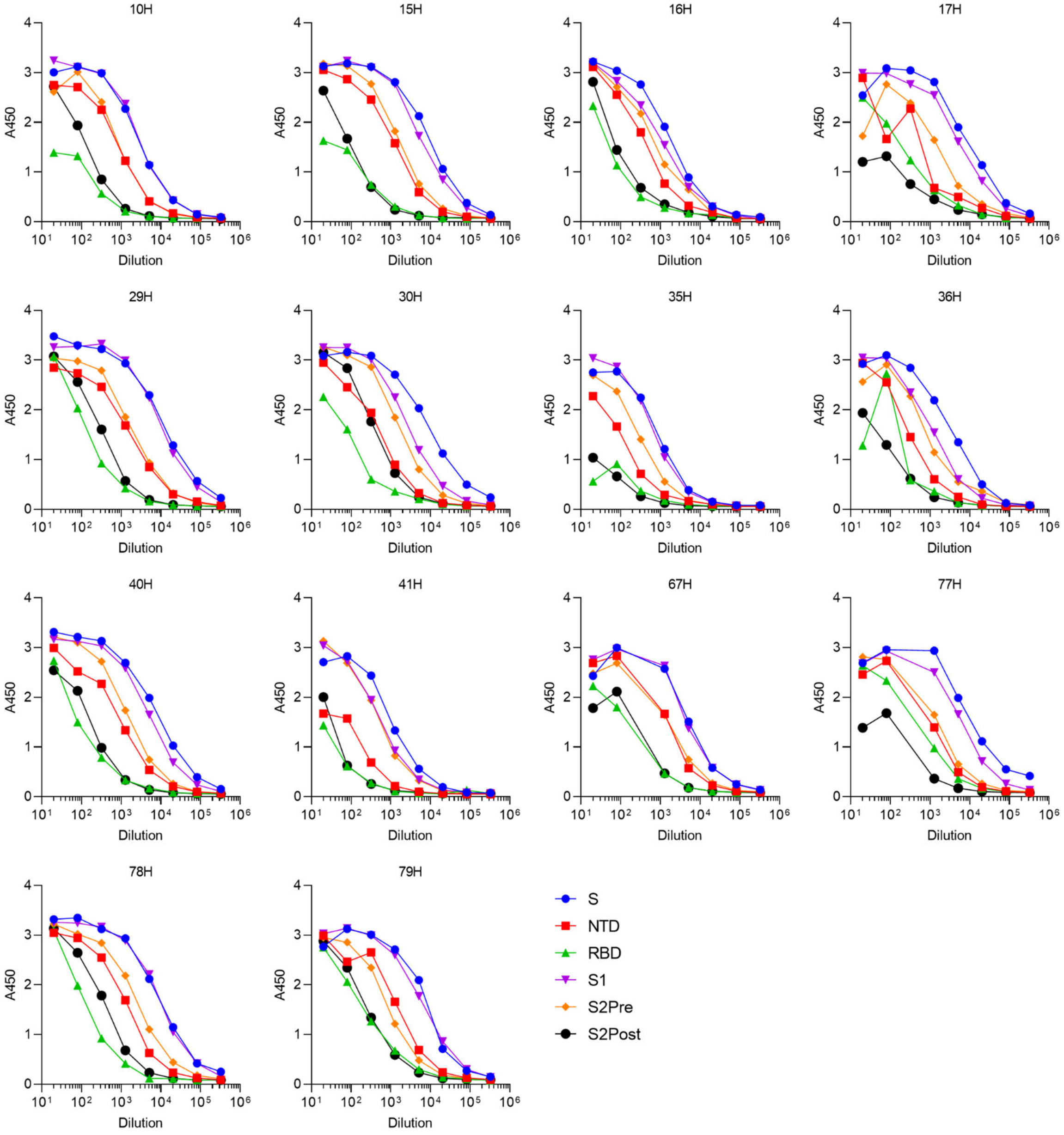

D 2x NVX-CoV2373

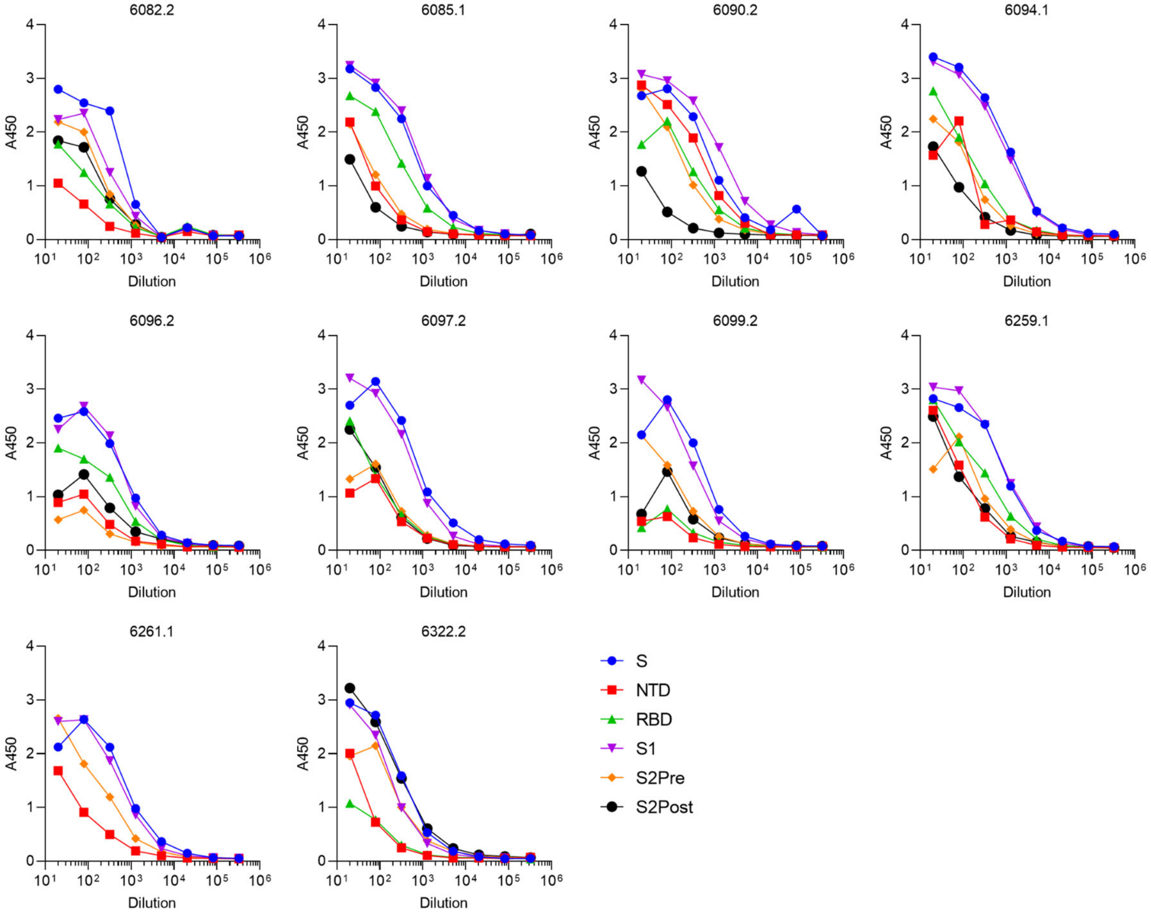

E 2x Ad26.COV2.S

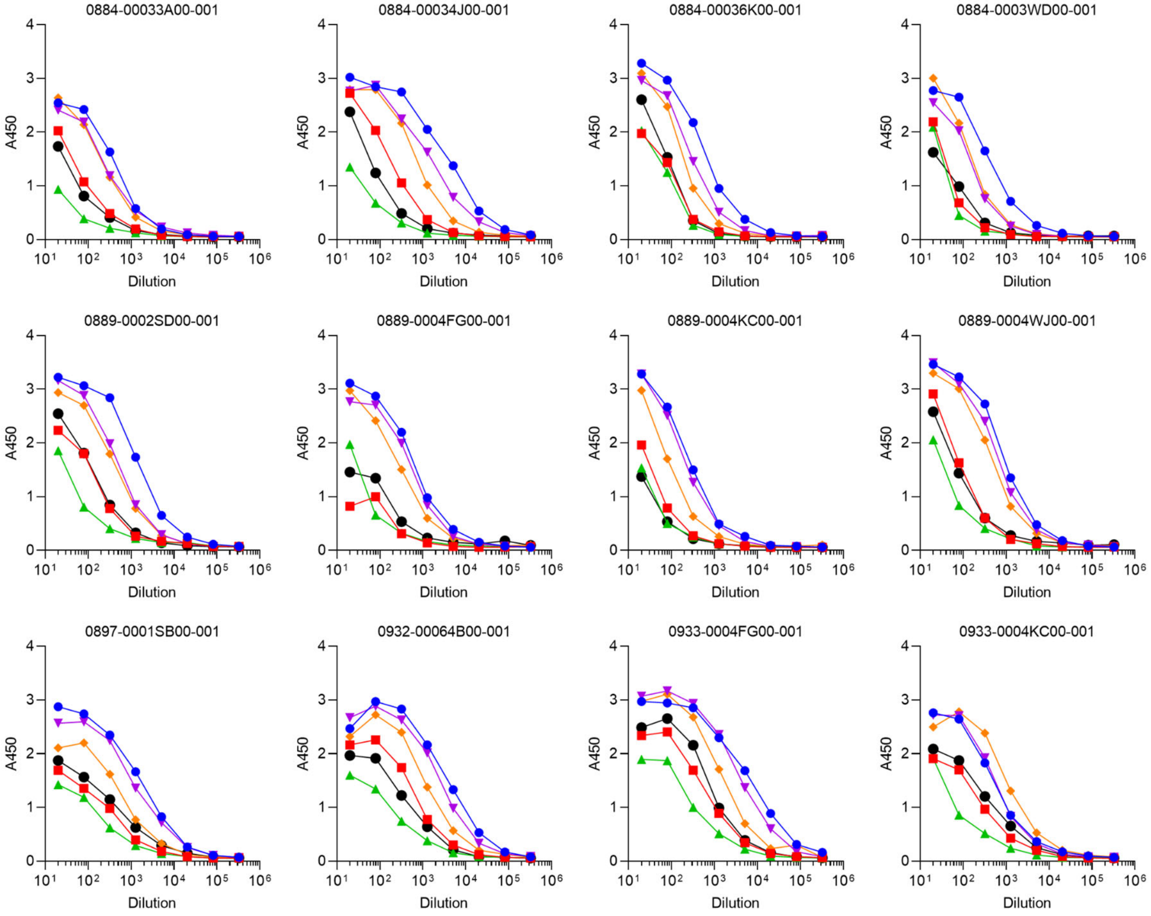

F 2x AZD1222

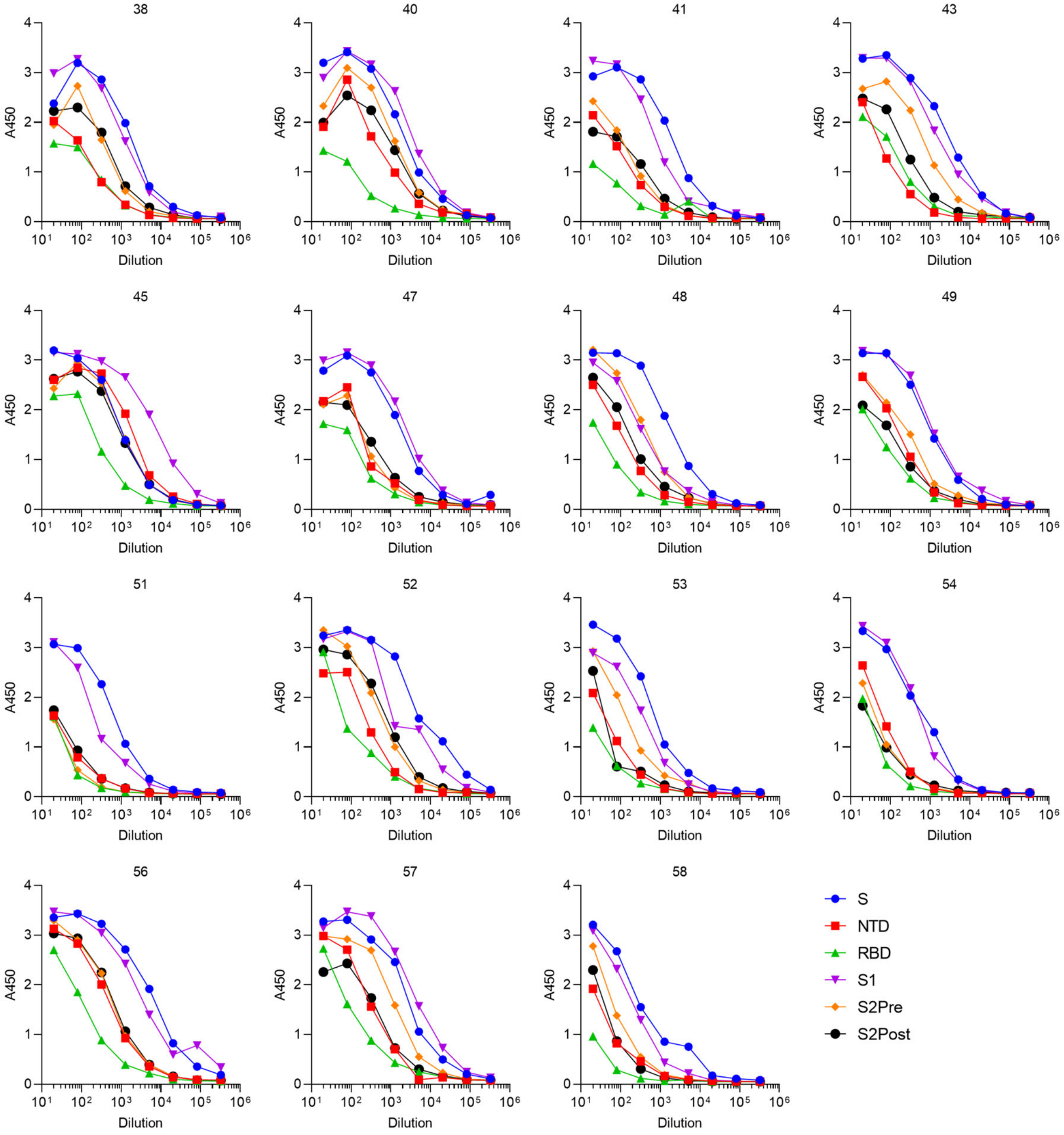

38
40
41
43
45
47
48
49
51
52
53
54
56
57
58
A450
Dilution
S
NTD
RBD
S1
S2Pre
S2Post

G 2x Sputnik V

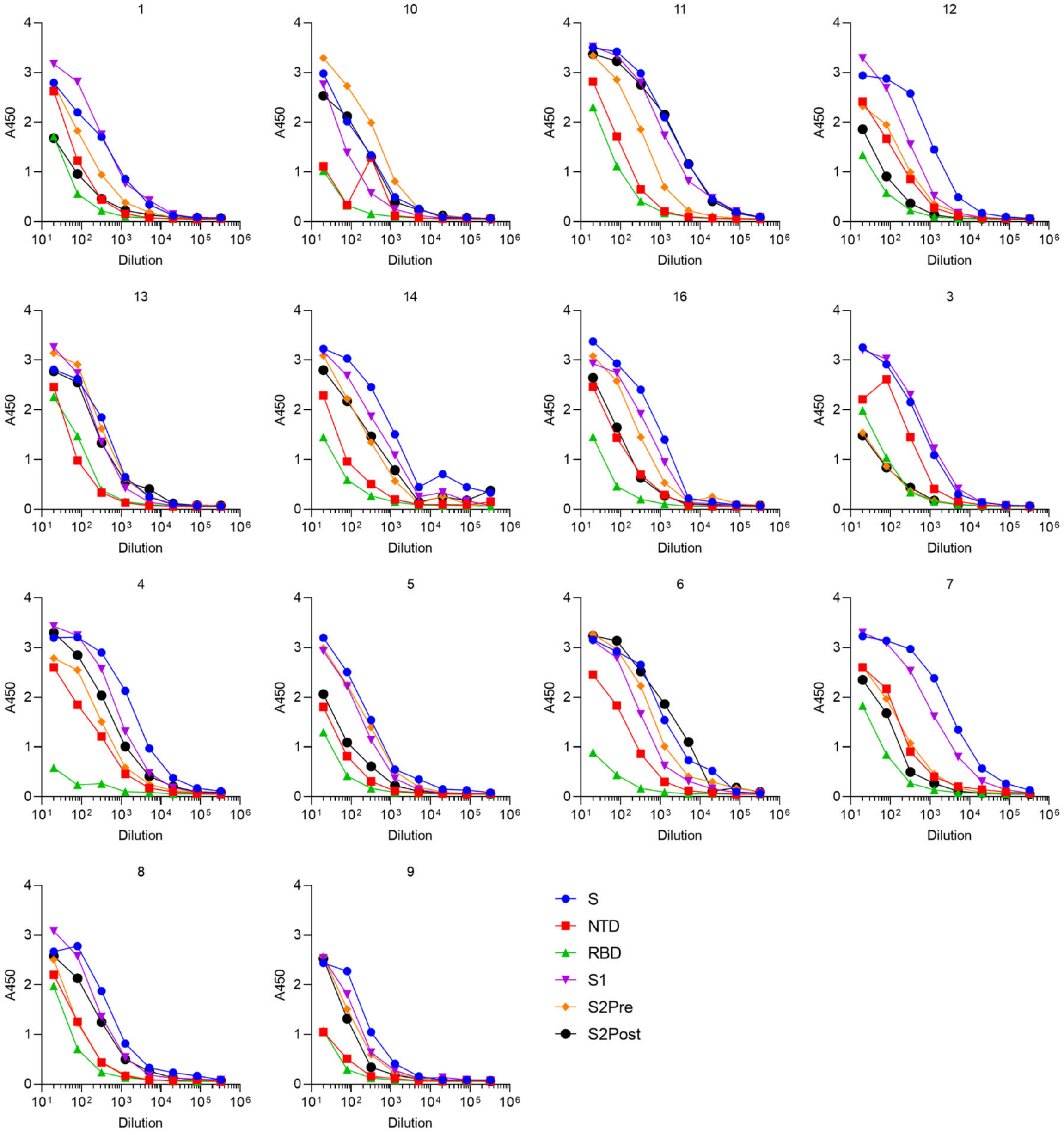

H 2x BBIBP-CorV

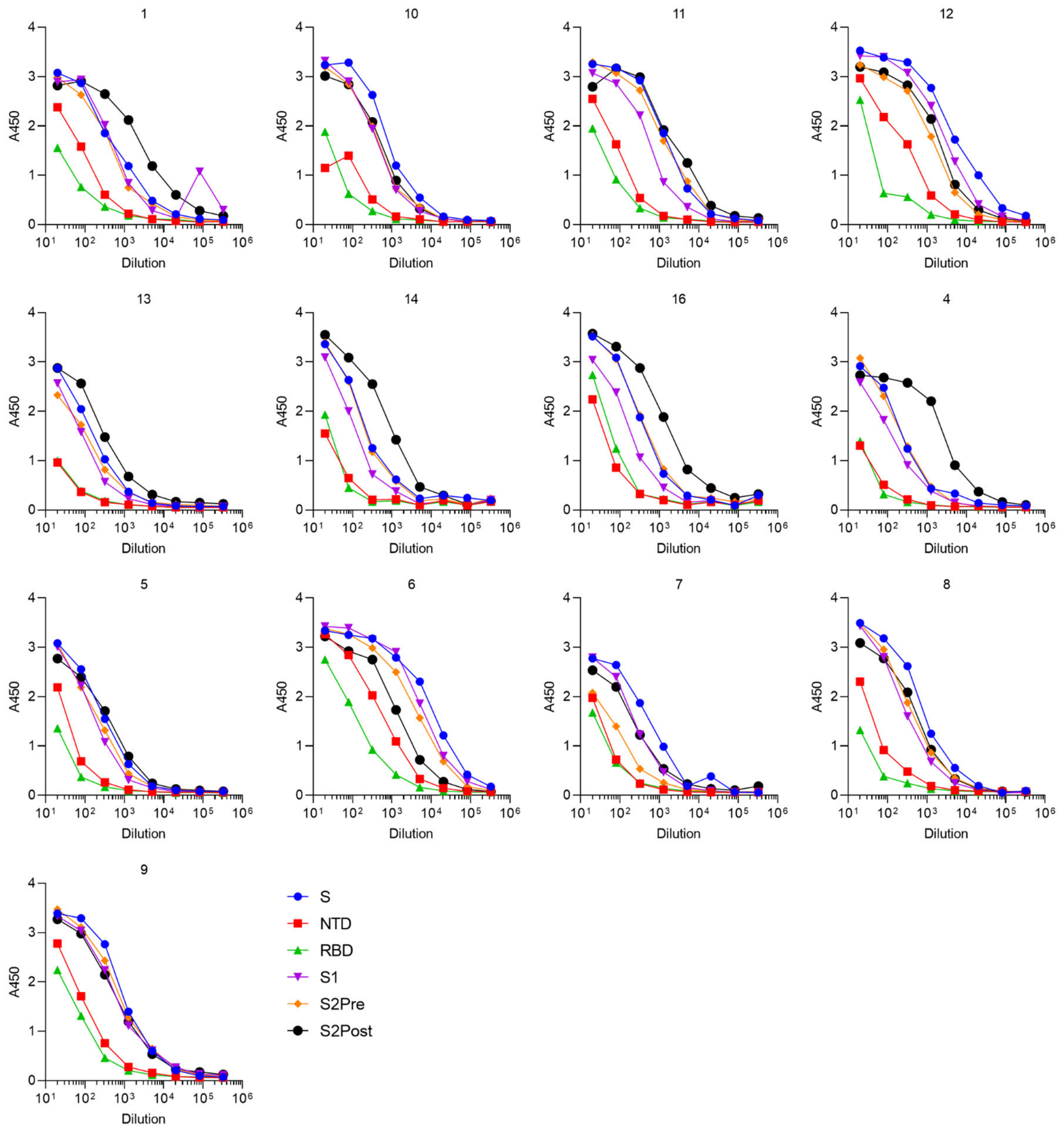

Figure S5. **Analysis of plasma antibody binding titers in previously infected subjects or vaccinees by ELISA.** (**A-H**) Dose-response curves of plasma antibody binding to the SARS-CoV-2 S, the N-terminal domain (NTD), the receptor-binding domain (RBD), the S_1 subunit, and the S_2 subunit in the prefusion and postfusion conformations. Samples were collected from individuals after SARS-CoV-2 infection (A) or vaccination with two doses of mRNA-1273 (B), BNT162b2 (C), Ad26.COV2.S (D), AZD1222 (E), Sputnik V (F), or BBIBP-CorV (E). Patient demographics can be found in Table S2. Curves are representative of at least two experimental replicates consisting of different batches of each antigen.

A 1x Infected 2x BNT162b2

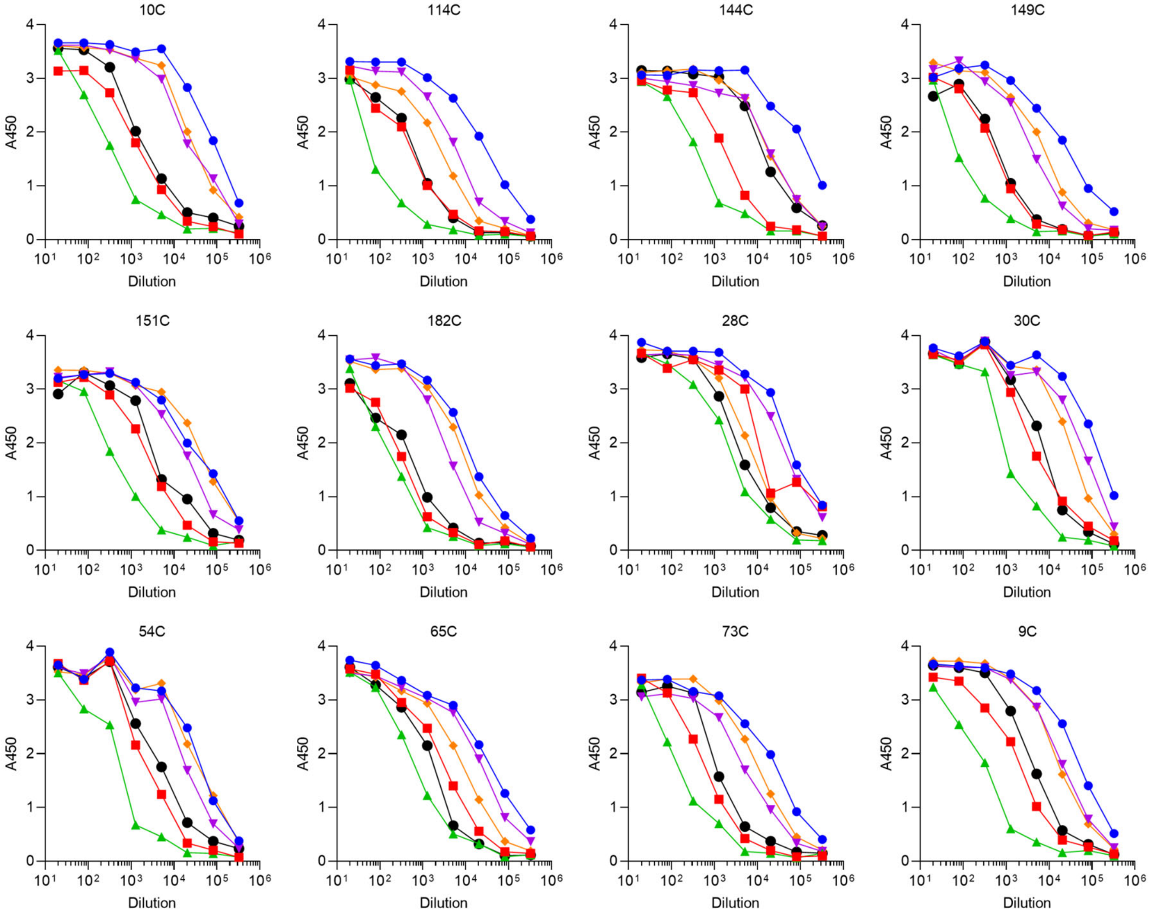

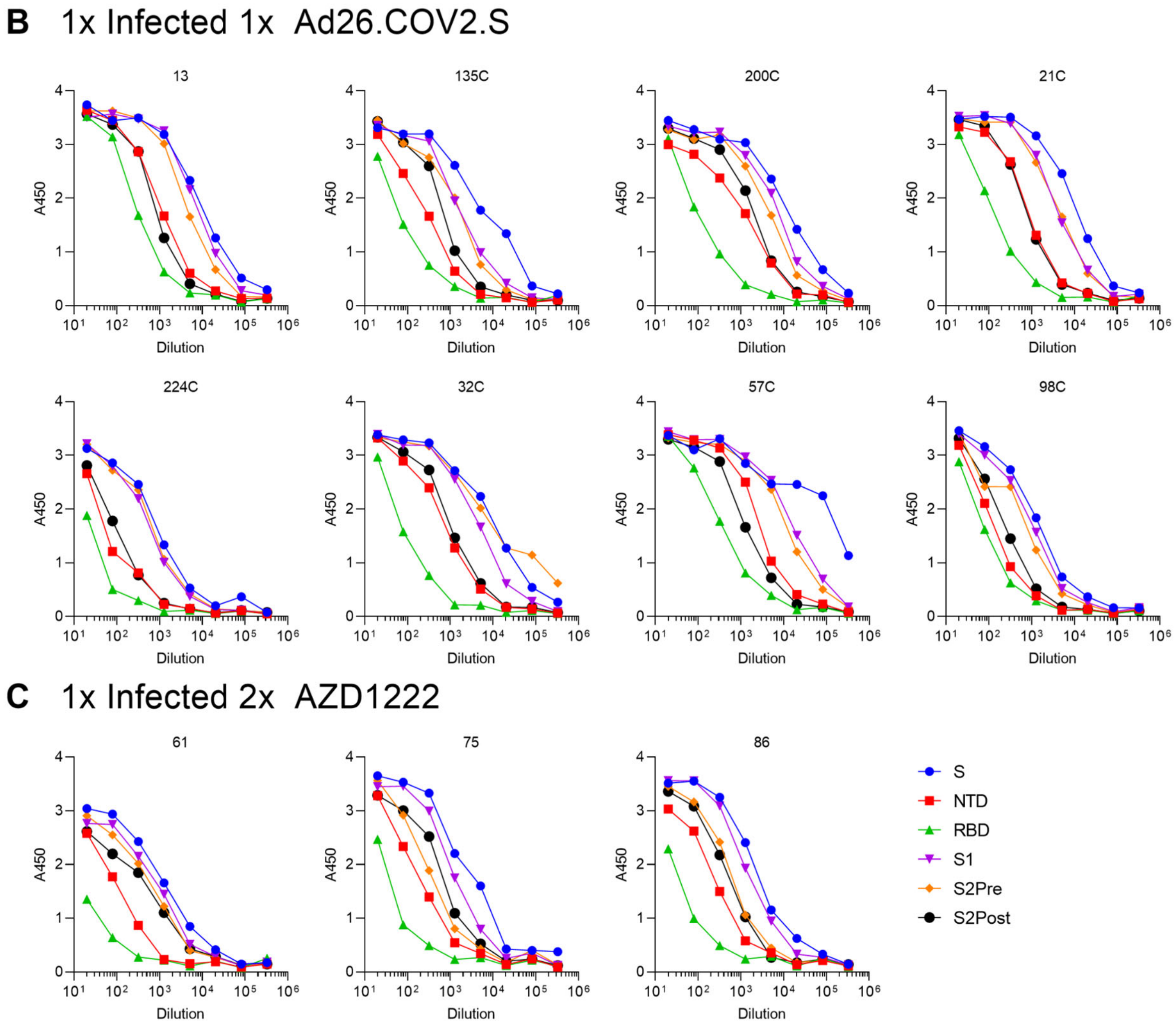

Figure S6. **Analysis of plasma antibody binding titers in previously infected subjects before and after vaccination by ELISA. A-C**, Dose-response curves of plasma IgG binding to the SARS-CoV-2 S, the N-terminal domain (NTD), the receptor-binding domain (RBD), the S_1 subunit, and the S_2 subunit in the prefusion and postfusion conformations. Samples were collected from individuals after SARS-CoV-2 infection (Wuhan-Hu-1-like strain) and vaccination with two doses of BNT162b2 (A), one dose of Ad26.COV2.S (B), or two doses of AZD1222 (C). Patient demographics can be found in Table S2. Curves are representative of at least two experimental replicates consisting of different batches of each antigen.

A 1x Infected

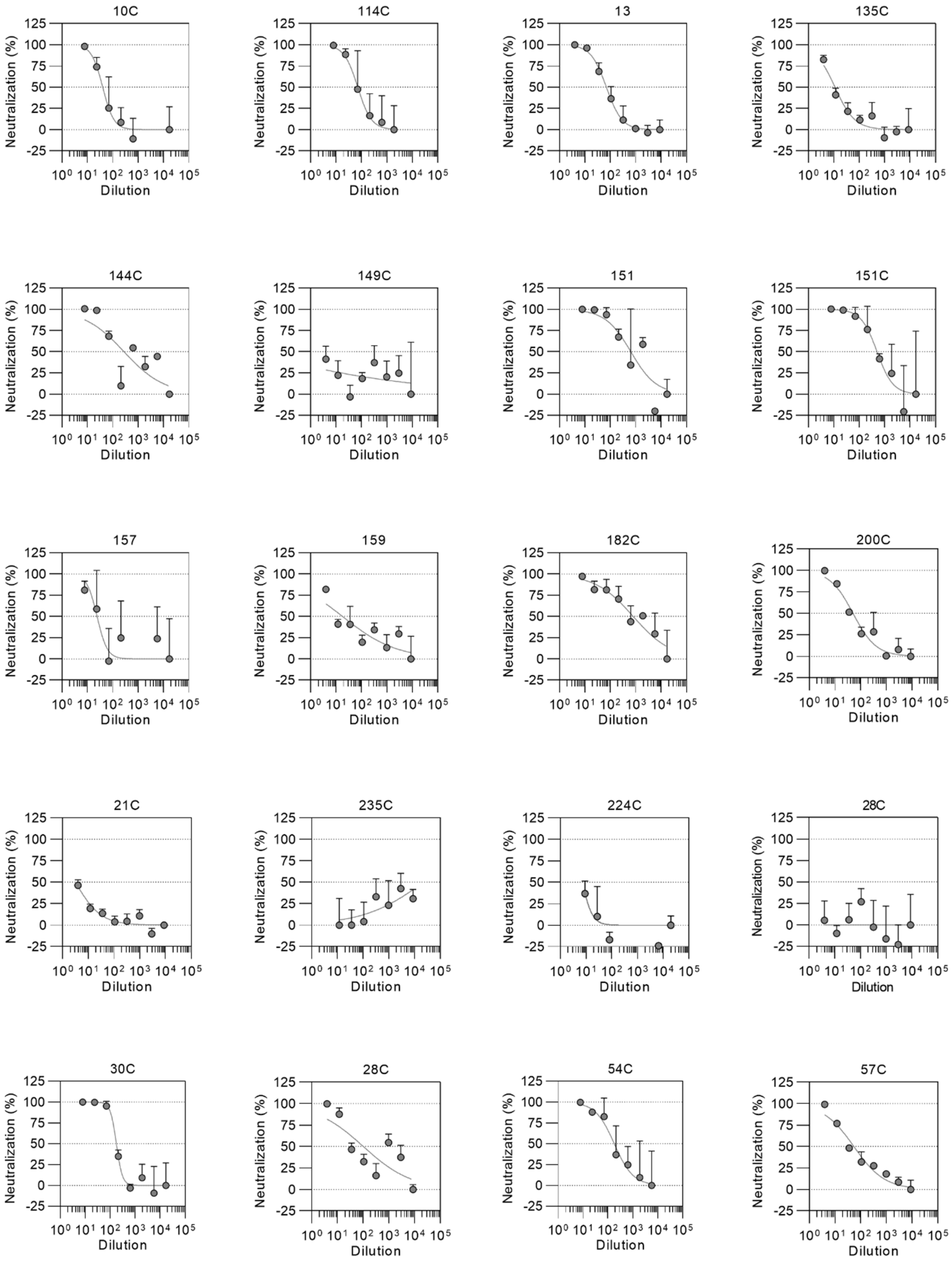

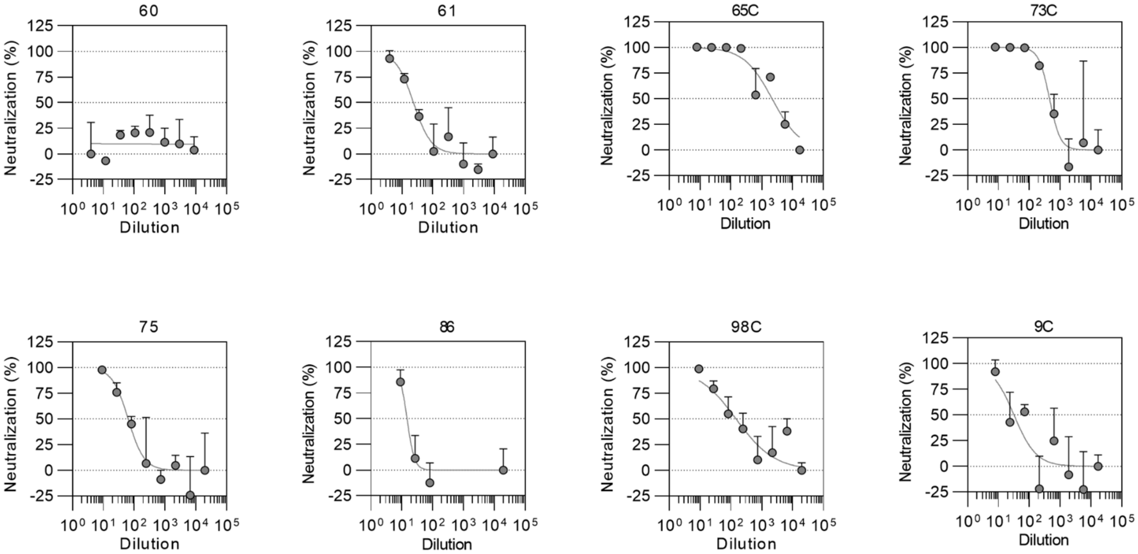
60
61
65C
73C
75
86
98C
9C
Neutralization (%)
Dilution
125
100
75
50
25
0
-25
10^0 10^1 10^2 10^3 10^4 10^5

B 2x mRNA-1273

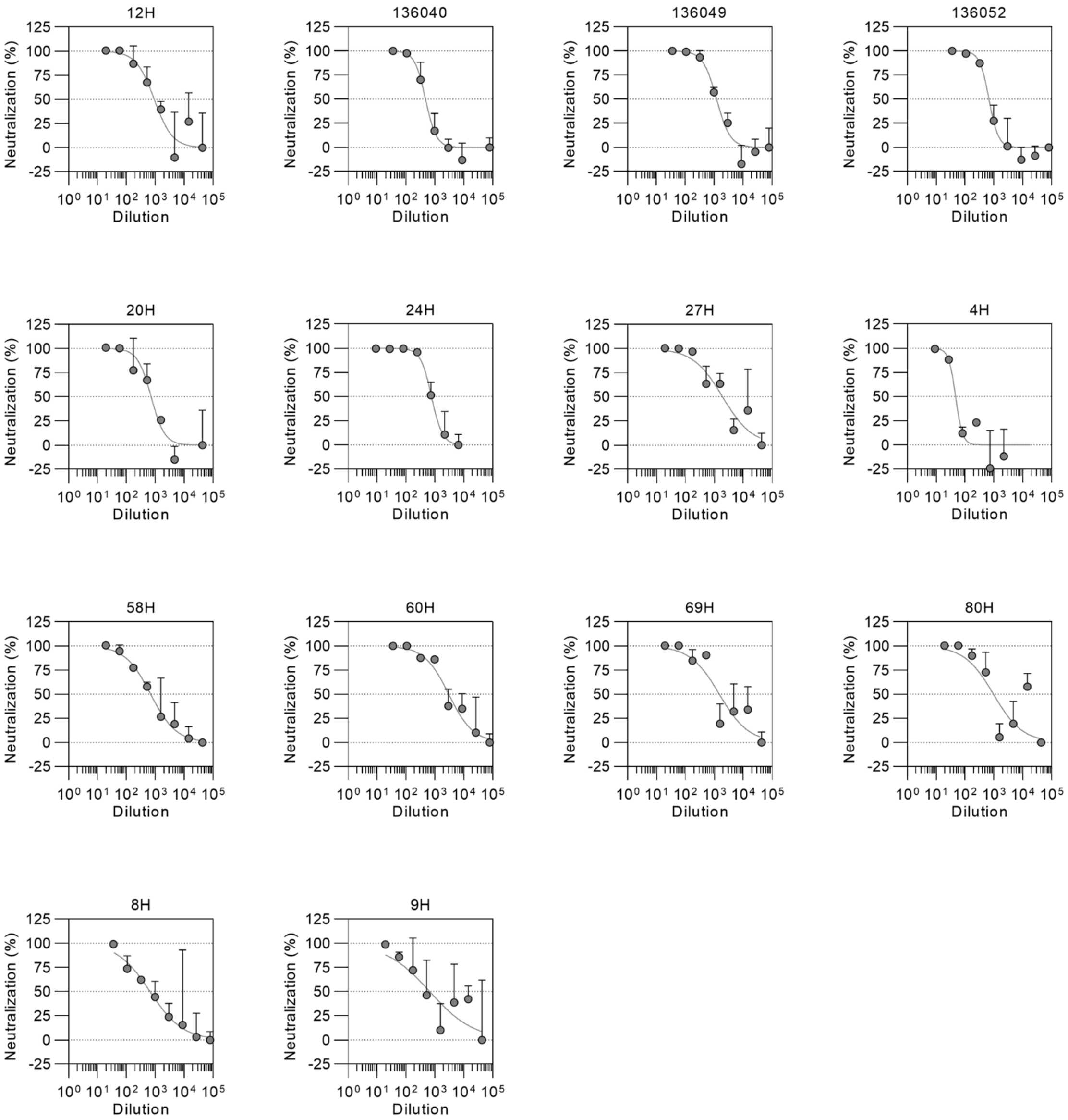

C 2x BNT162b2

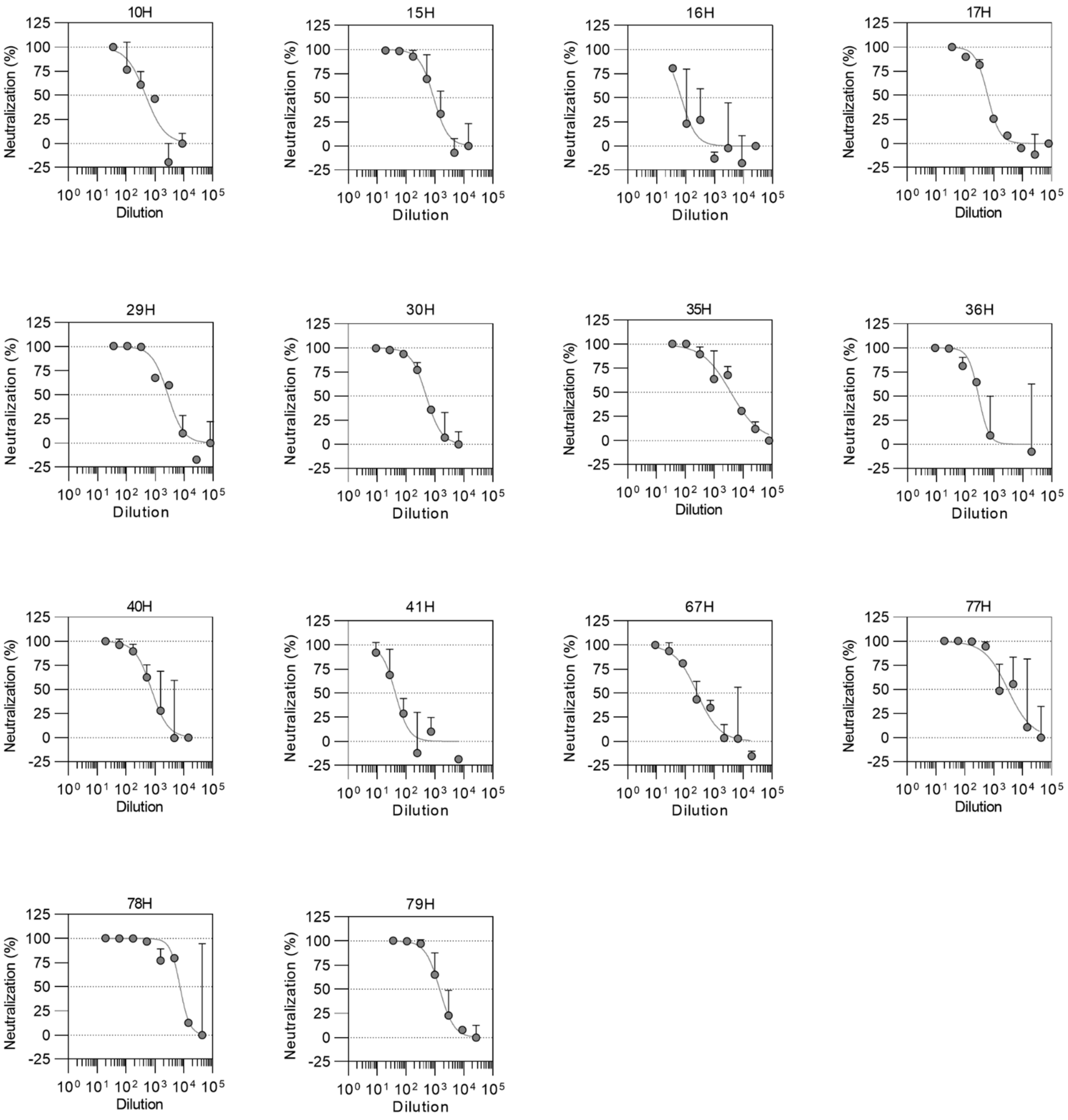

D 2x NVX-CoV2373

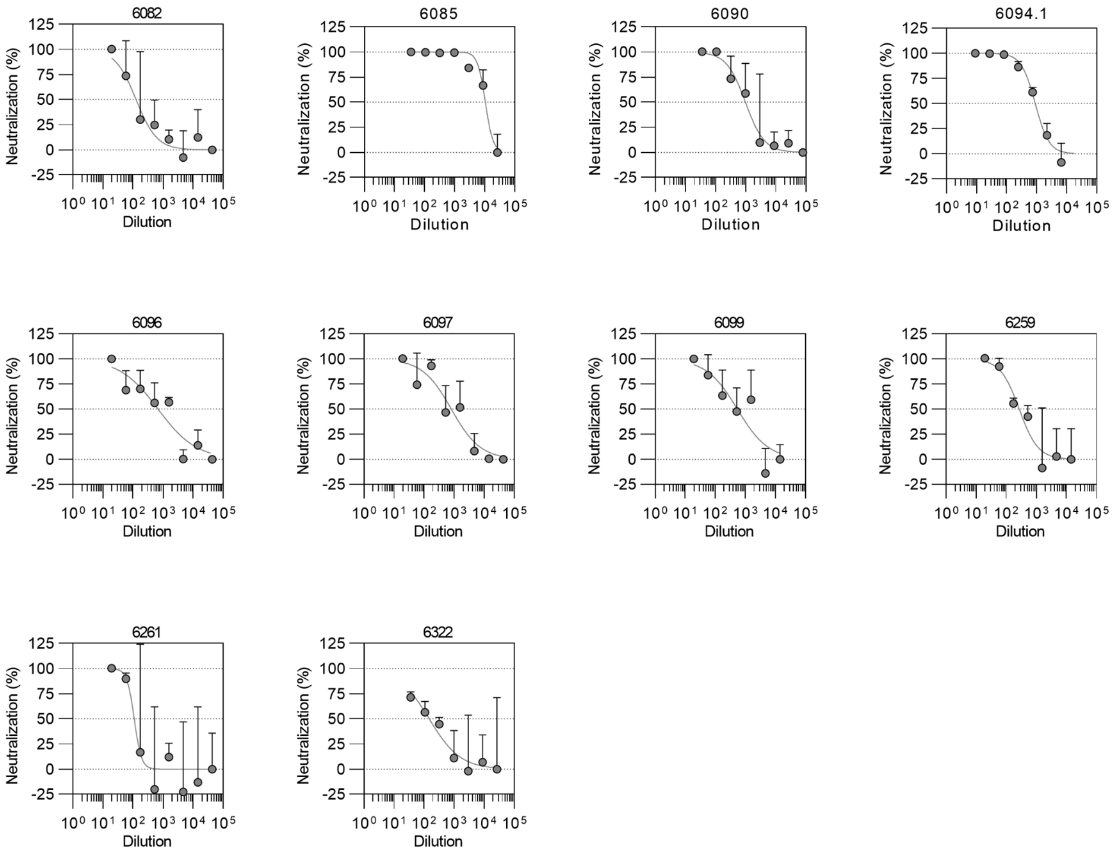

E 2x Ad26.COV2.S

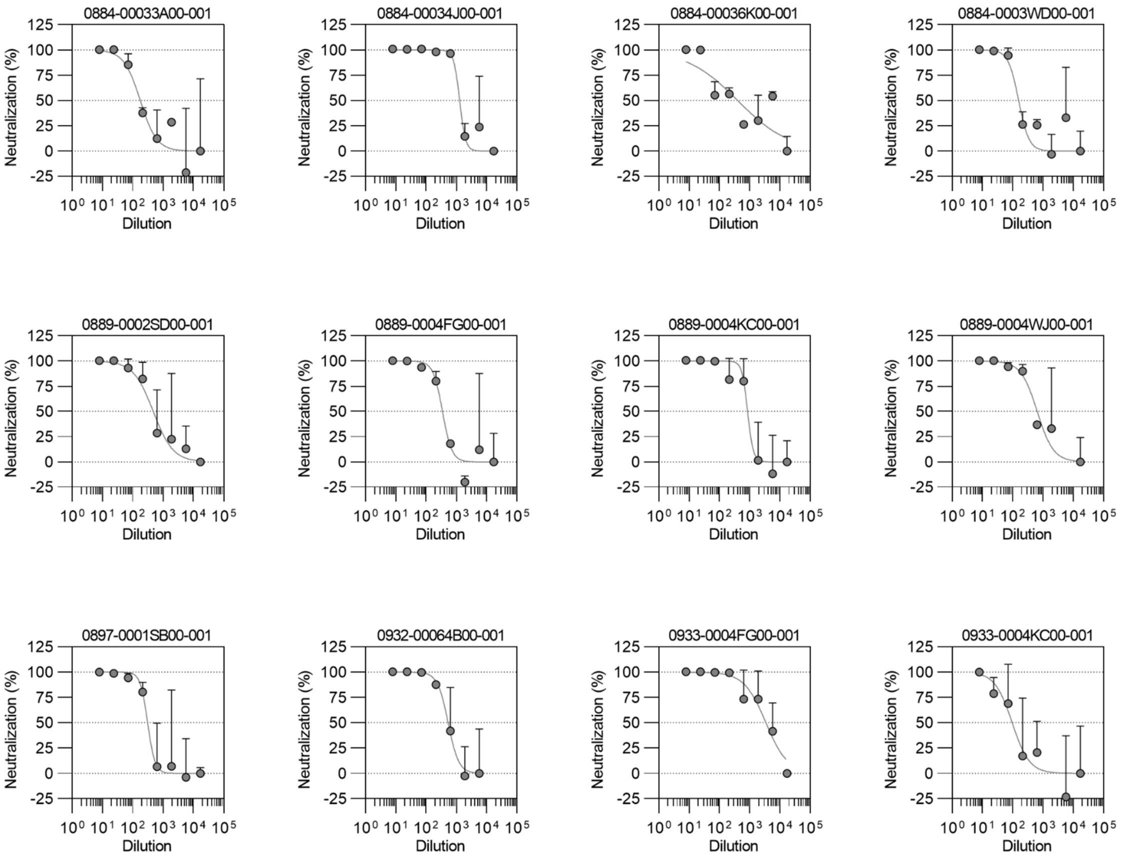

F 2x AZD1222

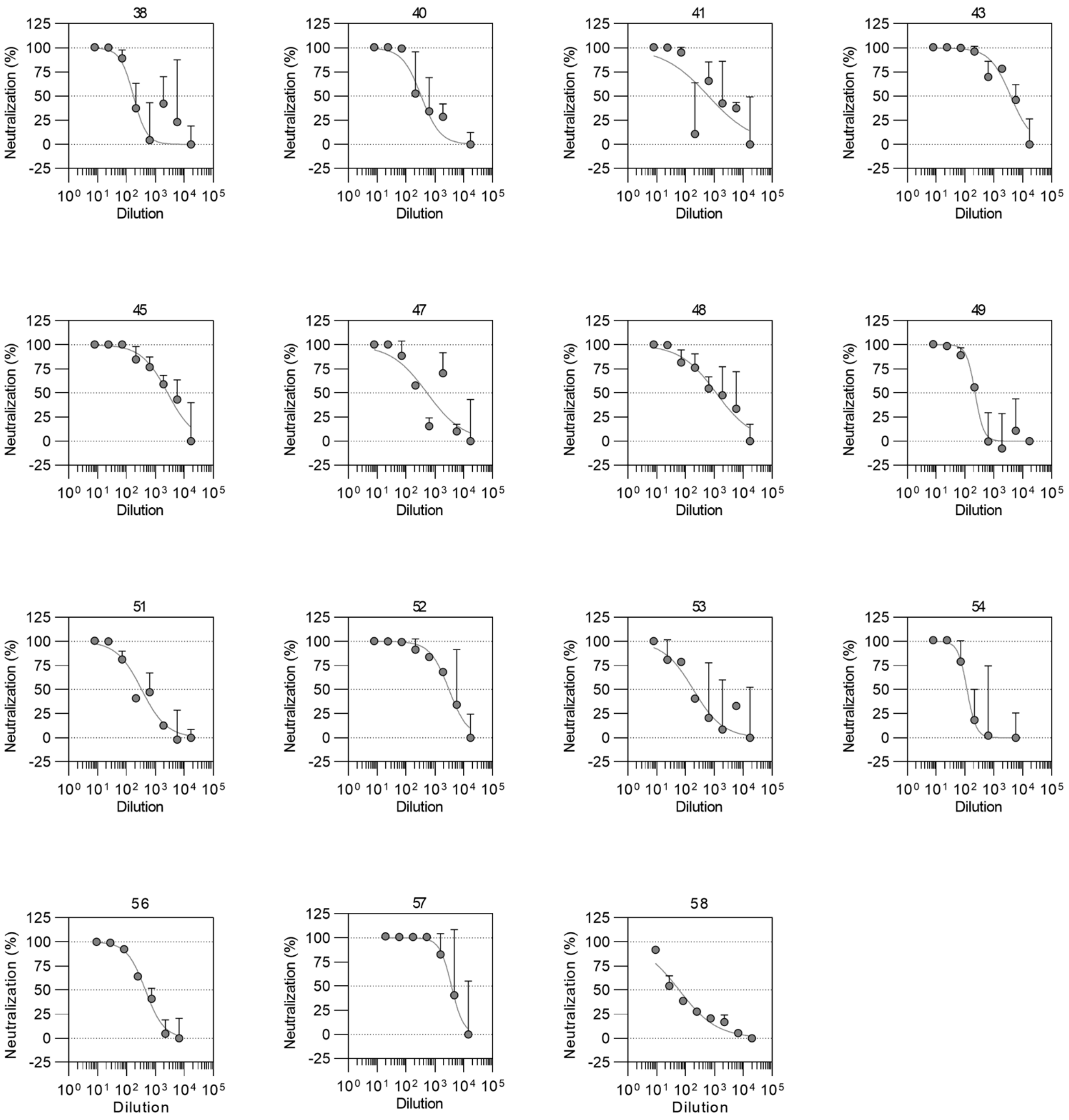
38
40
41
43
45
47
48
49
51
52
53
54
56
57
58
Neutralization (%)
125
100
75
50
25
0
-25
10^0 10^1 10^2 10^3 10^4 10^5
Dilution

G 2x Sputnik V

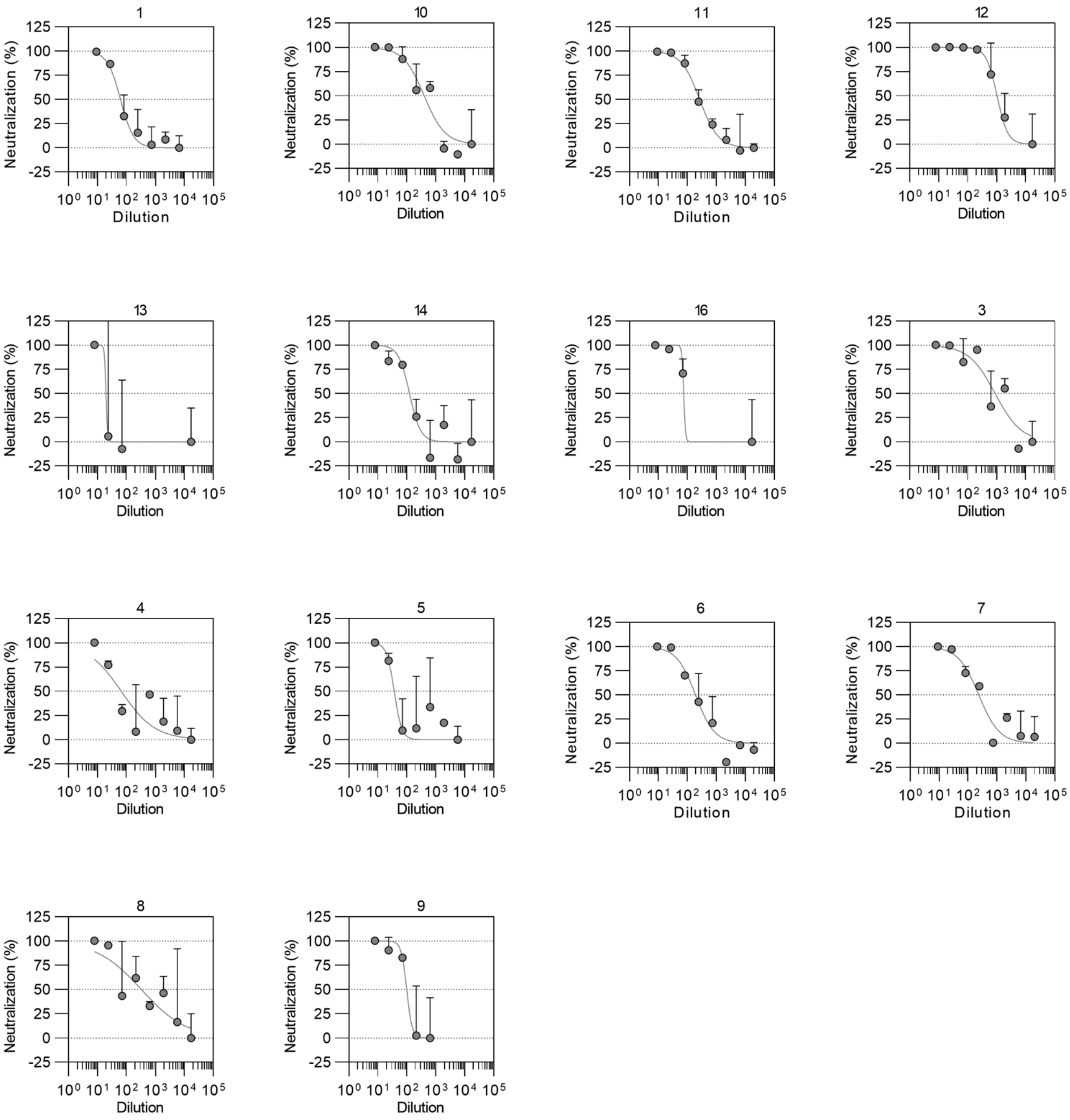

H 2x BBIBP-CorV

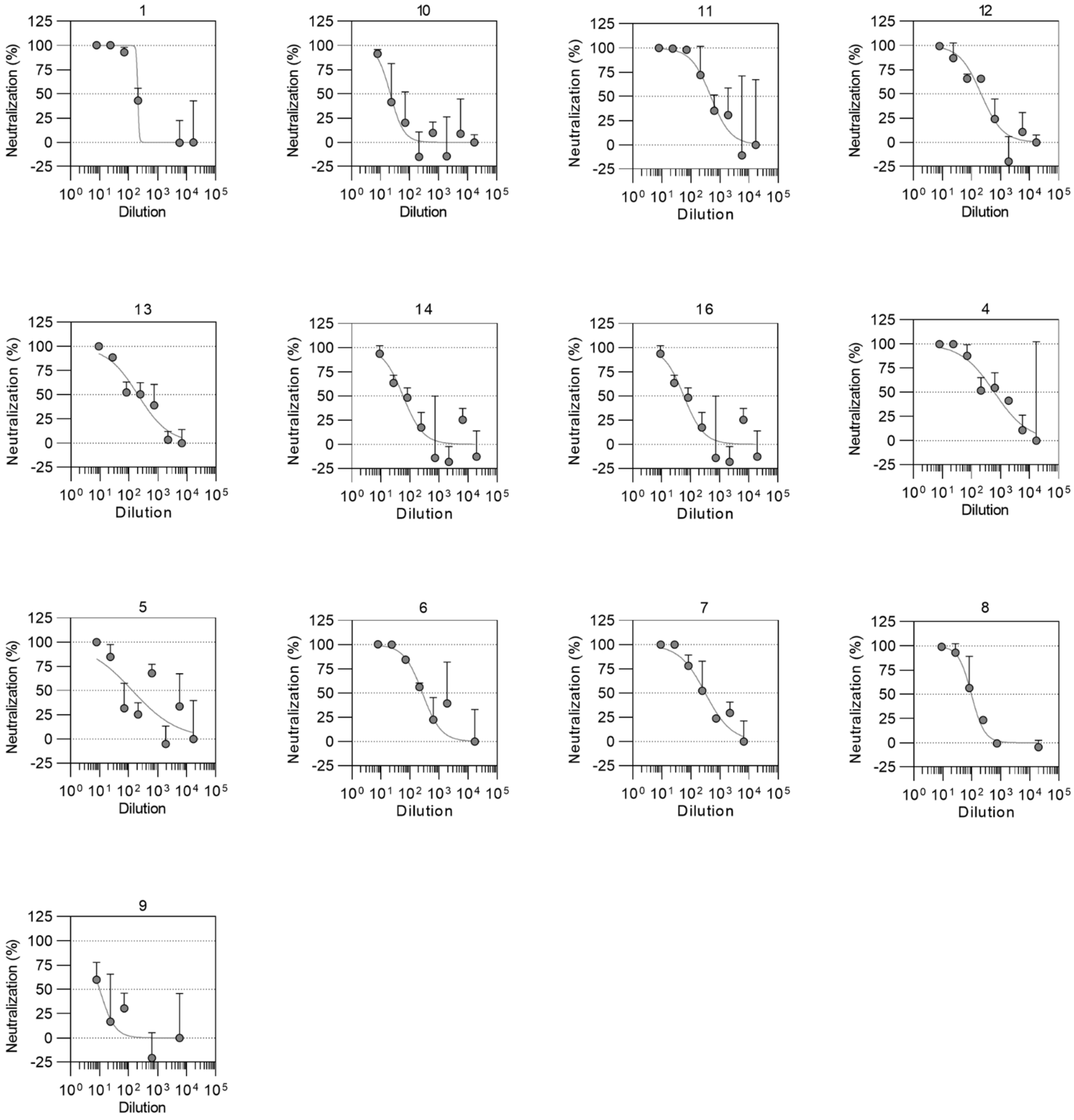

Figure S7. Evaluation of plasma neutralizing activity in previously infected subjects or vaccinees. A-E, Normalized dose-response neutralization curves using SARS-CoV-2 Wuhan-Hu-1/G614 S VSV pseudovirus and TMPRSS2-overexpressing VeroE6 target cells. Samples were collected from individuals after SARS-CoV-2 infection (A) or vaccination with two doses of mRNA-1273 (B), BNT162b2 (C), NVX-CoV2373 (D), Ad26.COV2.S (E), AZD1222 (F), Sputnik V (G), and BBIBP-CorV (H). Patient demographics can be found in Table S2. Points are the means of two-four replicates using the same pseudovirus and error bars are the geometric standard deviations. Curves are representative of at least two experimental replicates consisting of different batches of pseudovirus.

A 1x Infected 2x BNT162b2

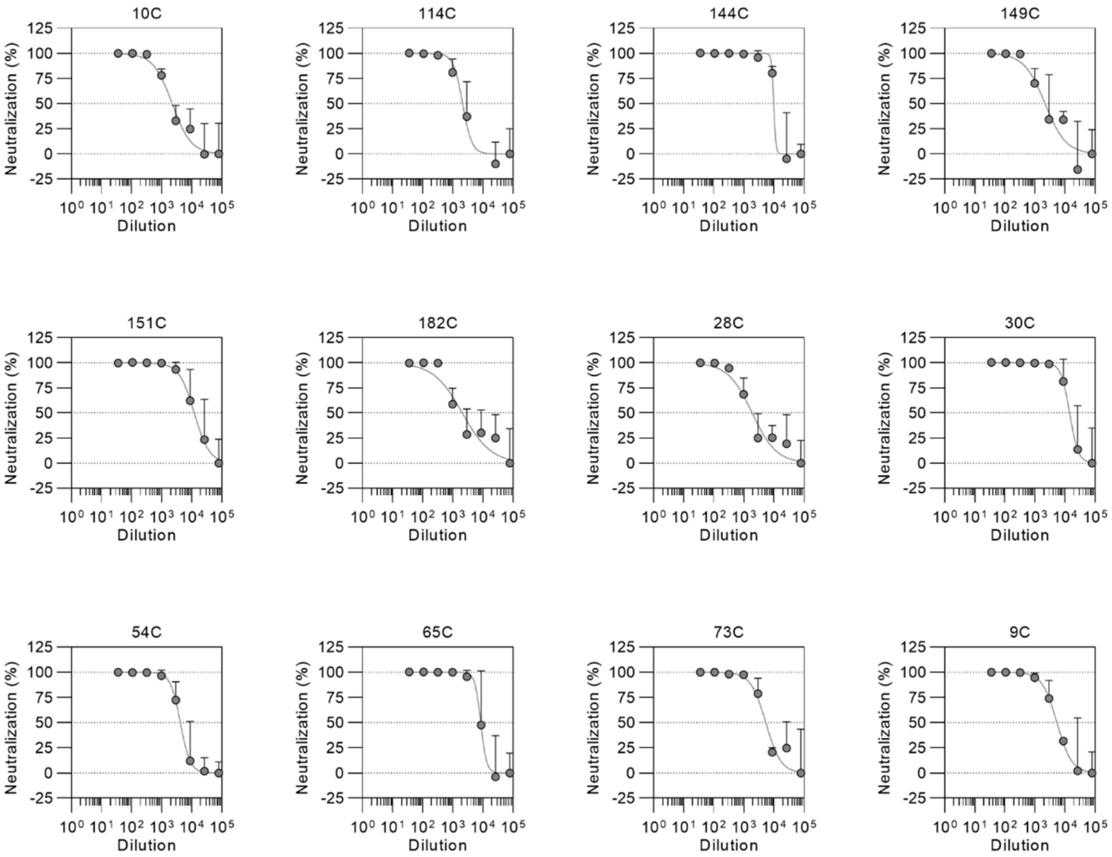

B 1x Infected 1x Ad26.COV2.S

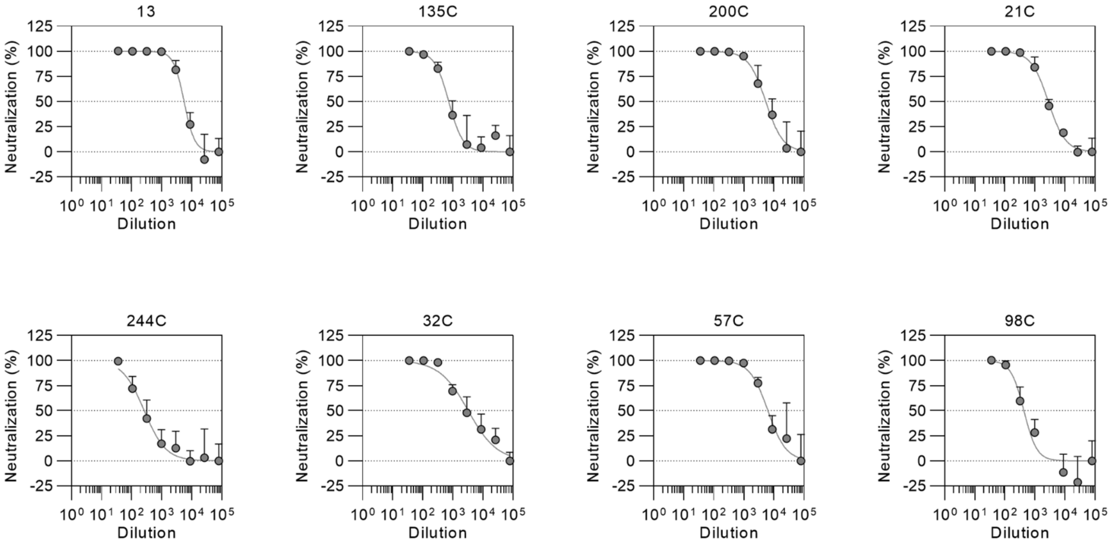

C 1x Infected 2x AZD1222

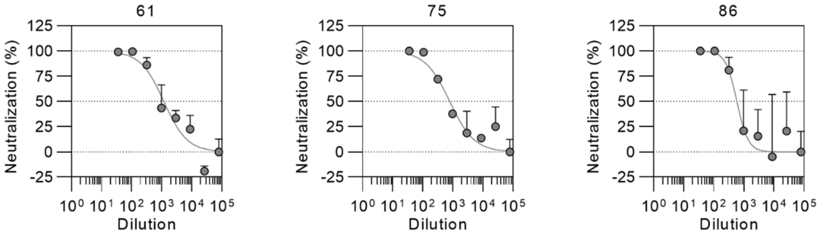

Figure S8. **Evaluation of plasma neutralizing activity in previously infected subjects before and after vaccination. A-C**, Normalized neutralization curves using SARS-CoV-2 Wuhan-Hu-1/G614 S VSV pseudovirus and TMPRSS2-overexpressing VeroE6 target cells. Samples were collected from individuals after SARS-CoV-2 infection (Wuhan-Hu-1-like strain) and vaccination with two doses of BNT162b2 (A), one dose of Ad26.COV2.S (B), or two doses of AZD1222 (C). Patient demographics can be found in Table S2. Points are the means of two-four replicates using the same pseudovirus and error bars are the geometric standard deviations. Curves are representative of at least two experimental replicates consisting of different batches of pseudovirus.

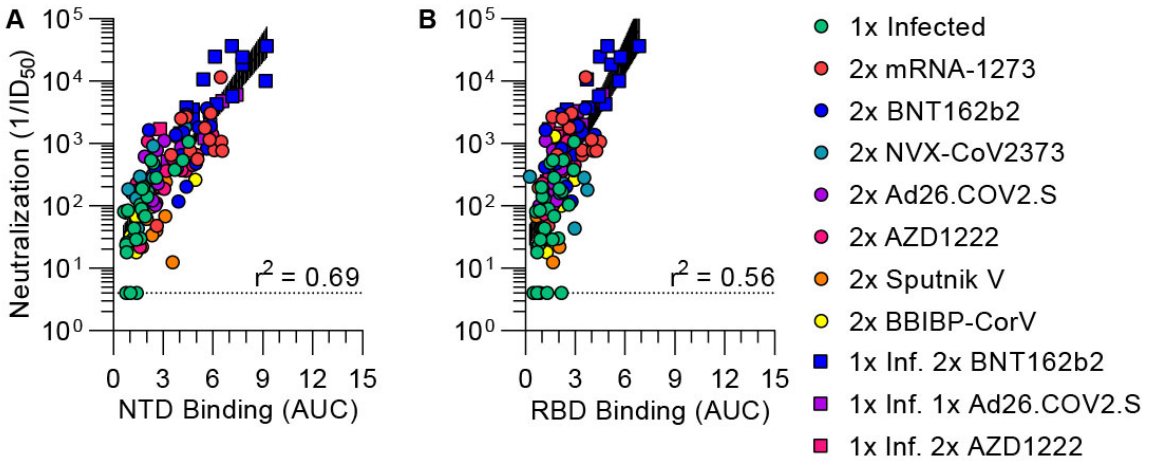

Figure S9. **A-B**, Correlation between NTD plasma IgG binding titers (A) or RBD plasma IgG binding titers (B) and neutralization titers. Binding AUCs were taken on log-transformed data and neutralization titers were log transformed before fitting to linear regression. The black shaded regions represent 95% confidence intervals.

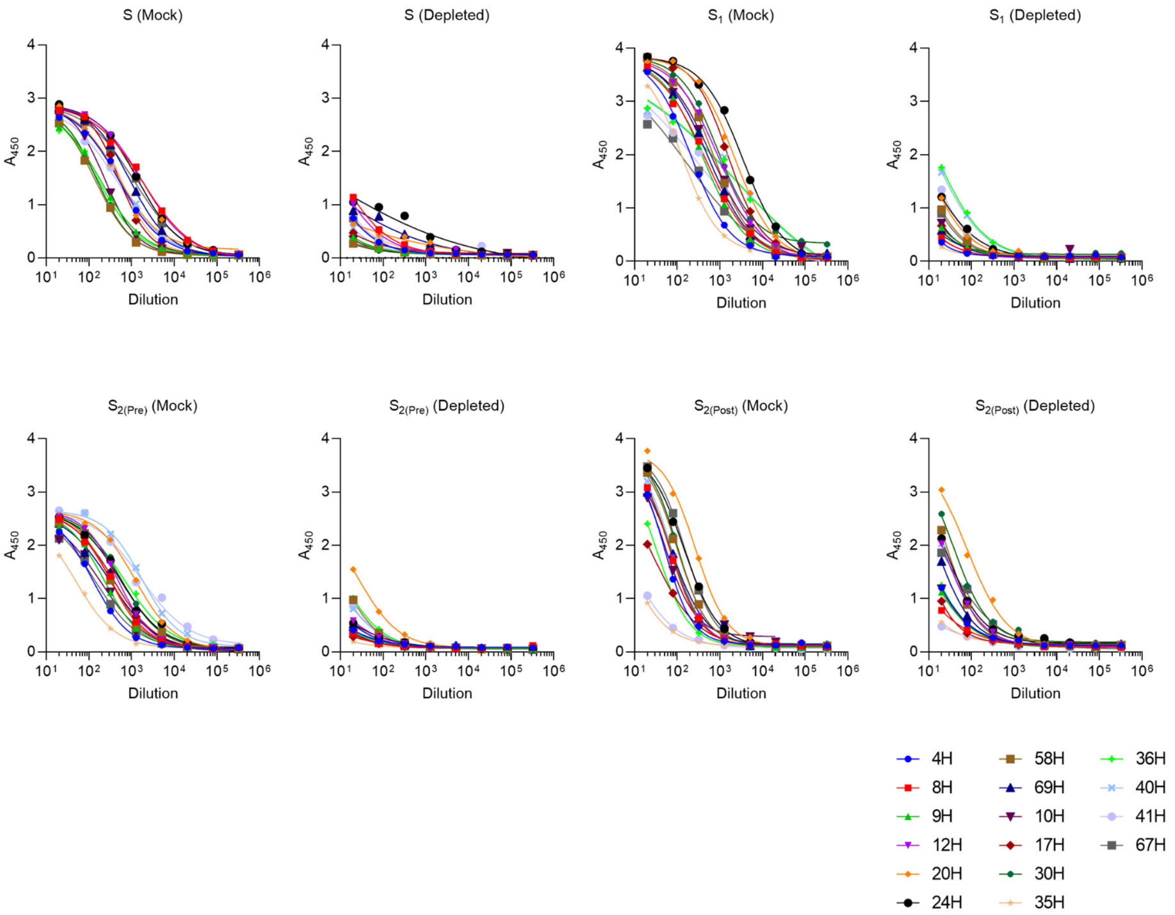

Figure S10. **Analysis of antibody binding titers in vaccinee plasma before or after antigen-specific depletion by ELISA.** Dose-response curves of plasma IgG binding to SARS-CoV-2 S, the S_1 subunit, or the S_2 subunit in the prefusion or postfusion conformation following mock depletion or depletion with each of these antigens. Samples were collected from individuals vaccinated with two doses of mRNA-1273 or BNT162b2. Curves are representative of at least two experimental replicates consisting of different batches of antigens and each color corresponds to one vaccinee.

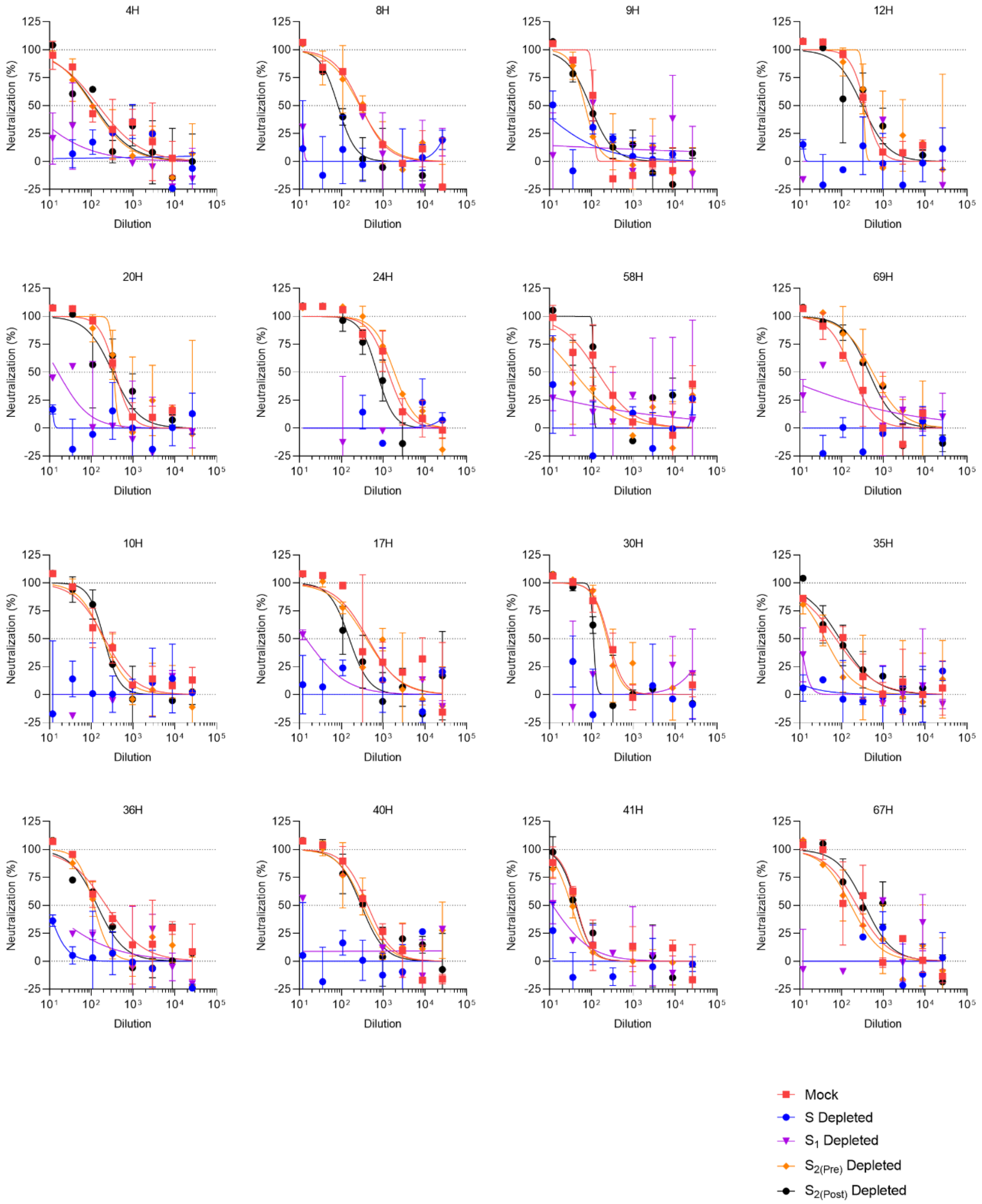

Figure S11. **Evaluation of plasma neutralizing activity in vaccinee plasma before or after antigen-specific depletion.** Normalized neutralization curves using SARS-CoV-2 Wuhan-Hu-1/G614 S VSV pseudovirus using VeroE6-TMPRSS2 target cells following mock depletion or depletion with S, the S_1 subunit, or the S_2 subunit in the prefusion or postfusion conformation. Samples were collected from individuals vaccinated with two doses of mRNA-1273 or BNT162b2. Patient demographics can be found in Table S2. Individual points are the mean of duplicates using the same antigen and error bars are the standard deviation of those duplicates. Curves are

representative of at least two experimental replicates consisting of different batches of antigens and pseudoviruses.

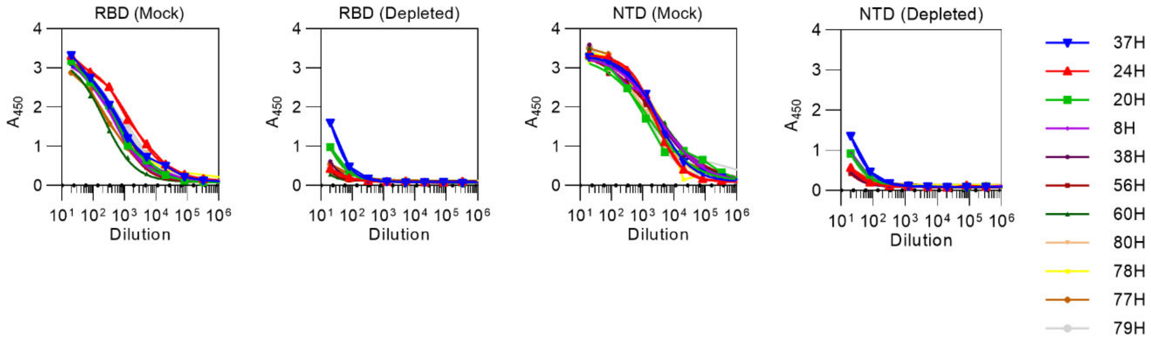

Figure S12. **Analysis of antibody binding titers in vaccinee plasma before or after antigen-specific depletion by ELISA.** Dose-response curves of plasma IgG binding to the RBD or the NTD following mock, RBD, or NTD depletion. Samples were collected from individuals vaccinated with two doses of mRNA-1273 or BNT162b12. Patient demographics can be found in Table S2. Curves are representative of at least two experimental replicates consisting of different batches of antigen and each color corresponds to one vaccinee.

A G614

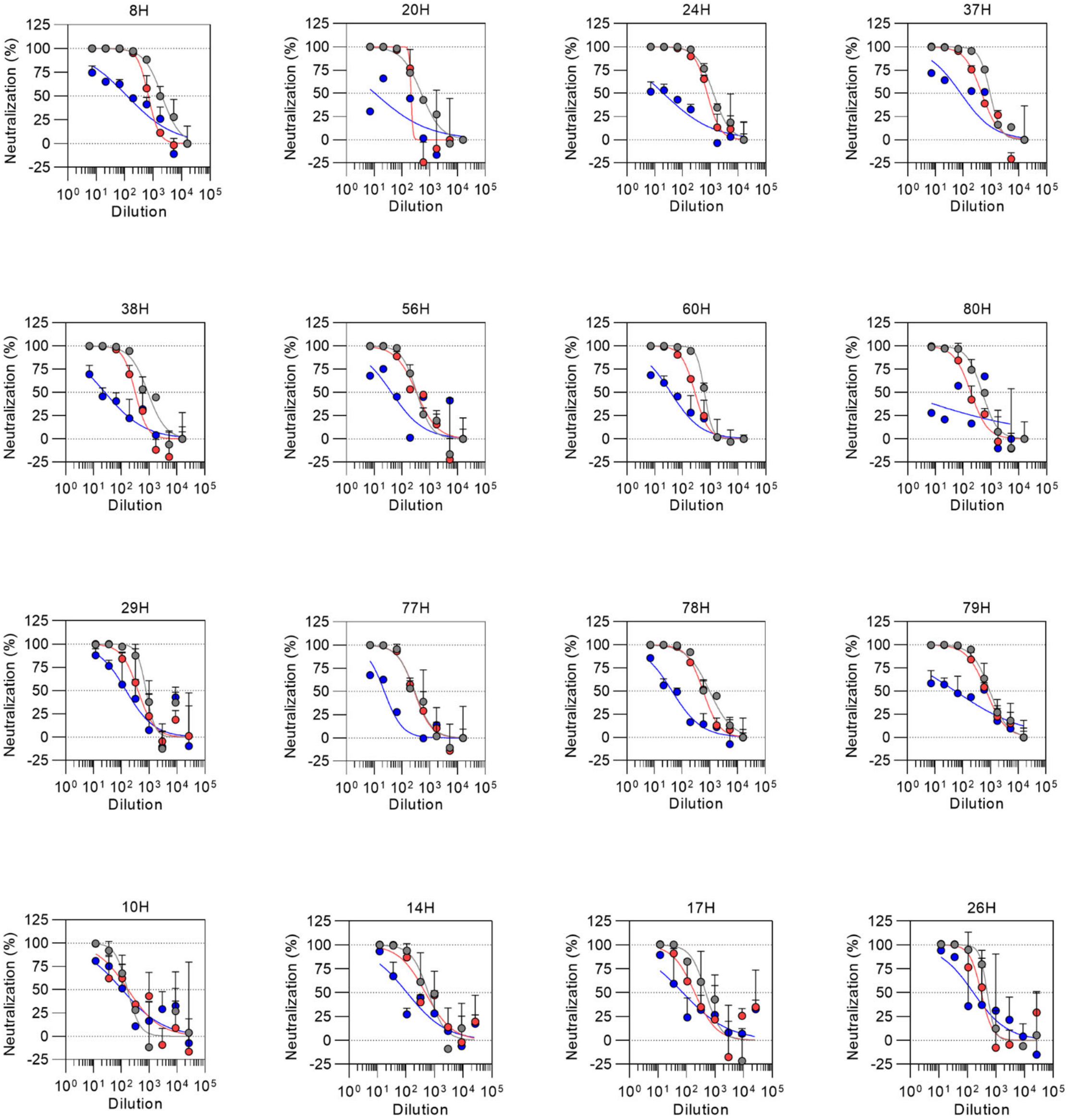
8H
20H
24H
37H
38H
56H
60H
80H
29H
77H
78H
79H
10H
14H
17H
26H
Neutralization (%)
Dilution
125
100
75
50
25
0
-25
10⁰ 10¹ 10² 10³ 10⁴ 10⁵

B Alpha

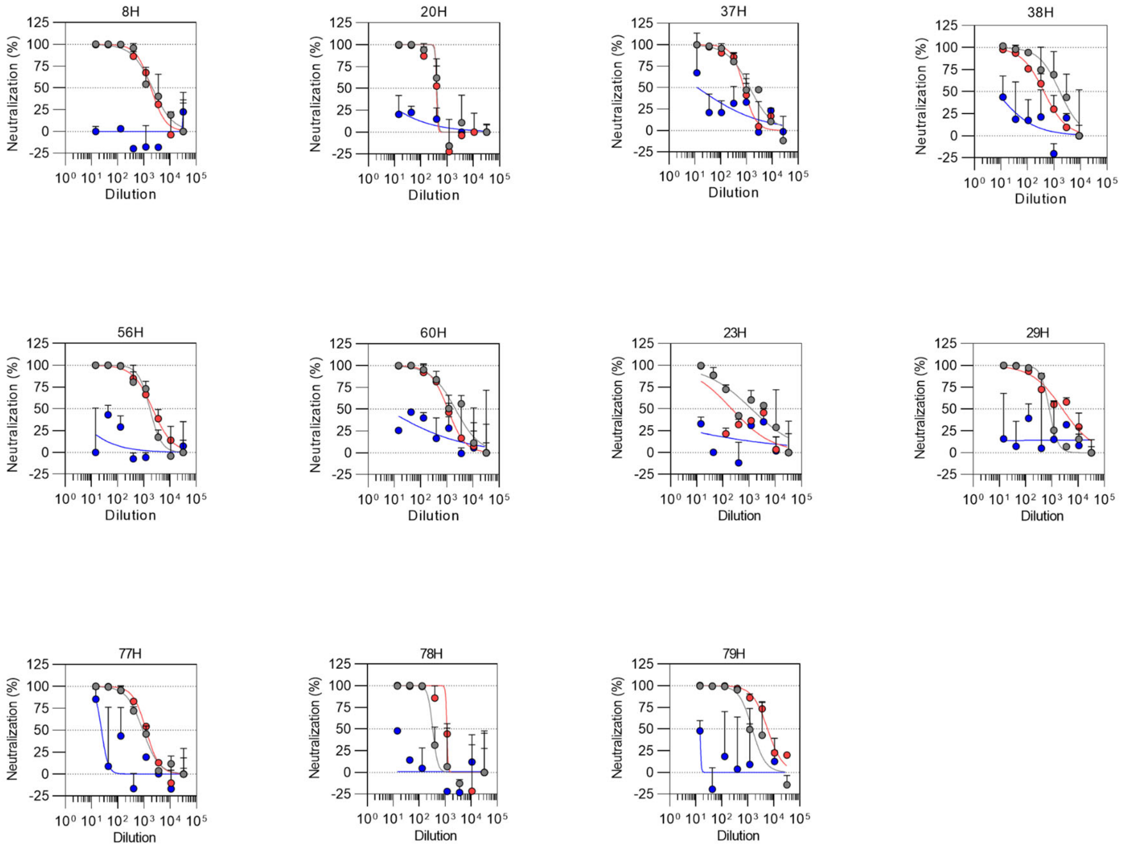

8H
20H
37H
38H
56H
60H
23H
29H
77H
78H
79H
Neutralization (%)
Dilution
125
100
75
50
25
0
-25
10^0 10^1 10^2 10^3 10^4 10^5

C Beta

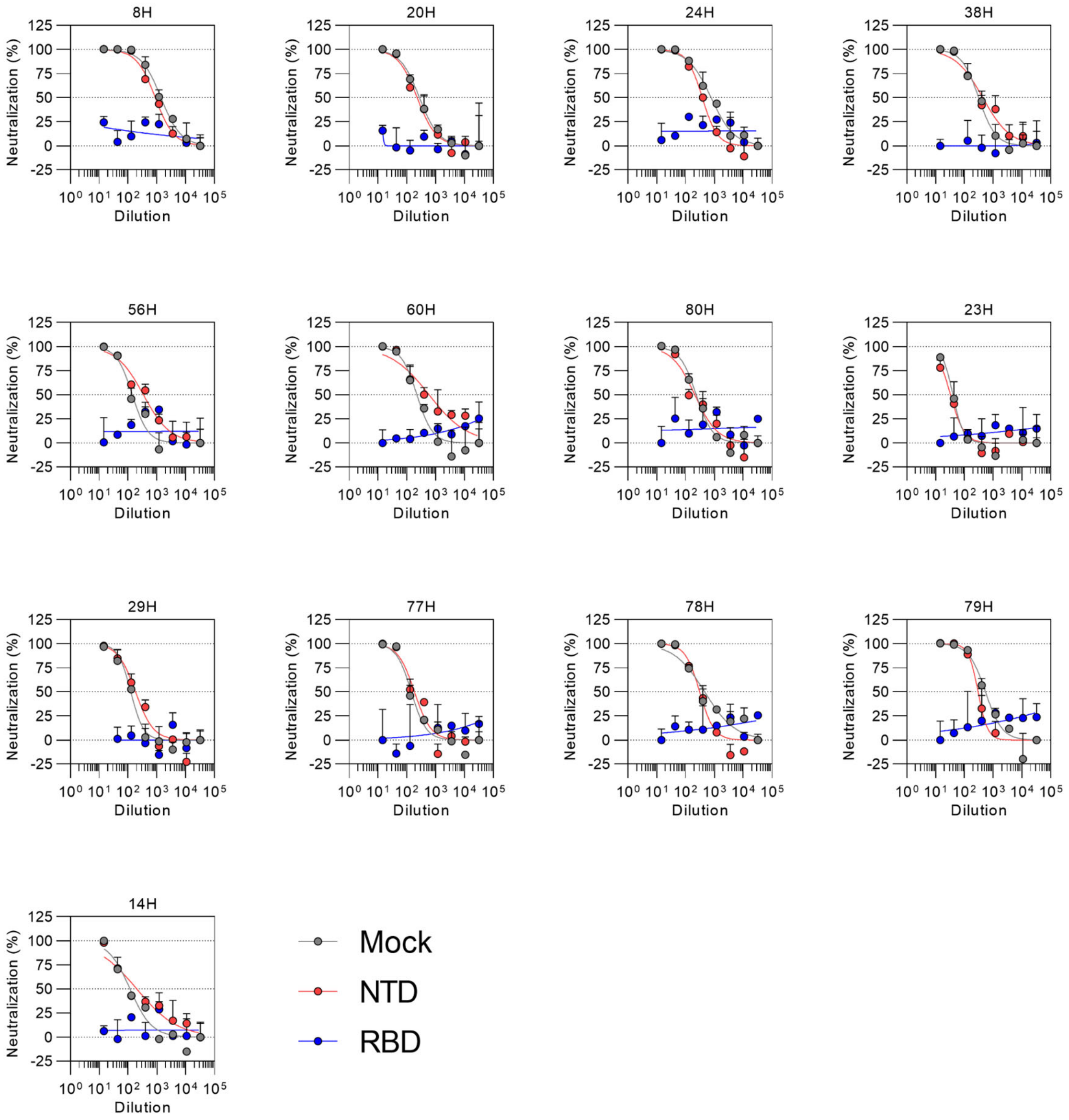
8H
20H
24H
38H
56H
60H
80H
23H
29H
77H
78H
79H
14H
Neutralization (%)
Dilution
Mock
NTD
RBD

D Delta

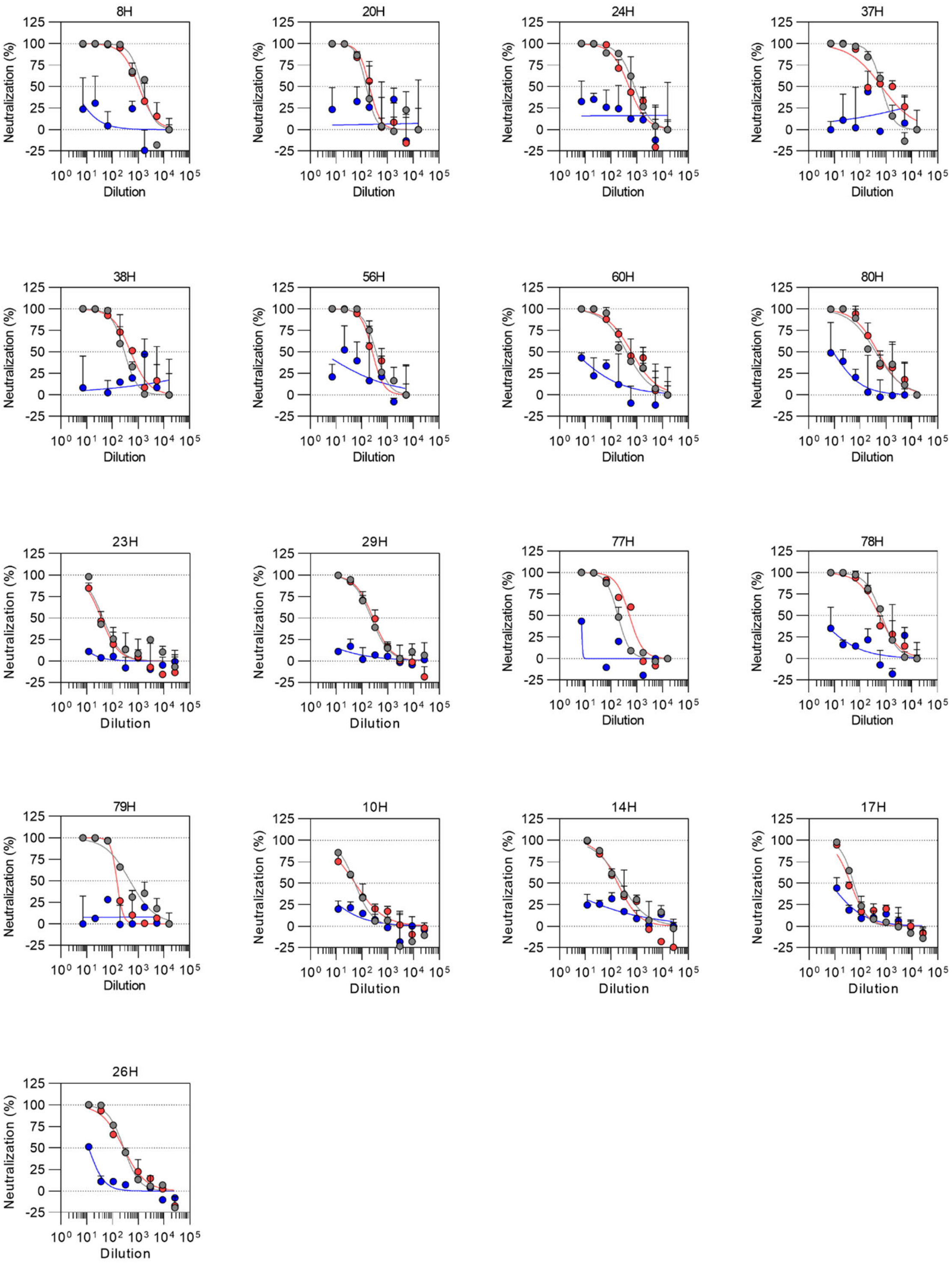

8H
20H
24H
37H
38H
56H
60H
80H
23H
29H
77H
78H
79H
10H
14H
17H
26H
Neutralization (%)
Dilution

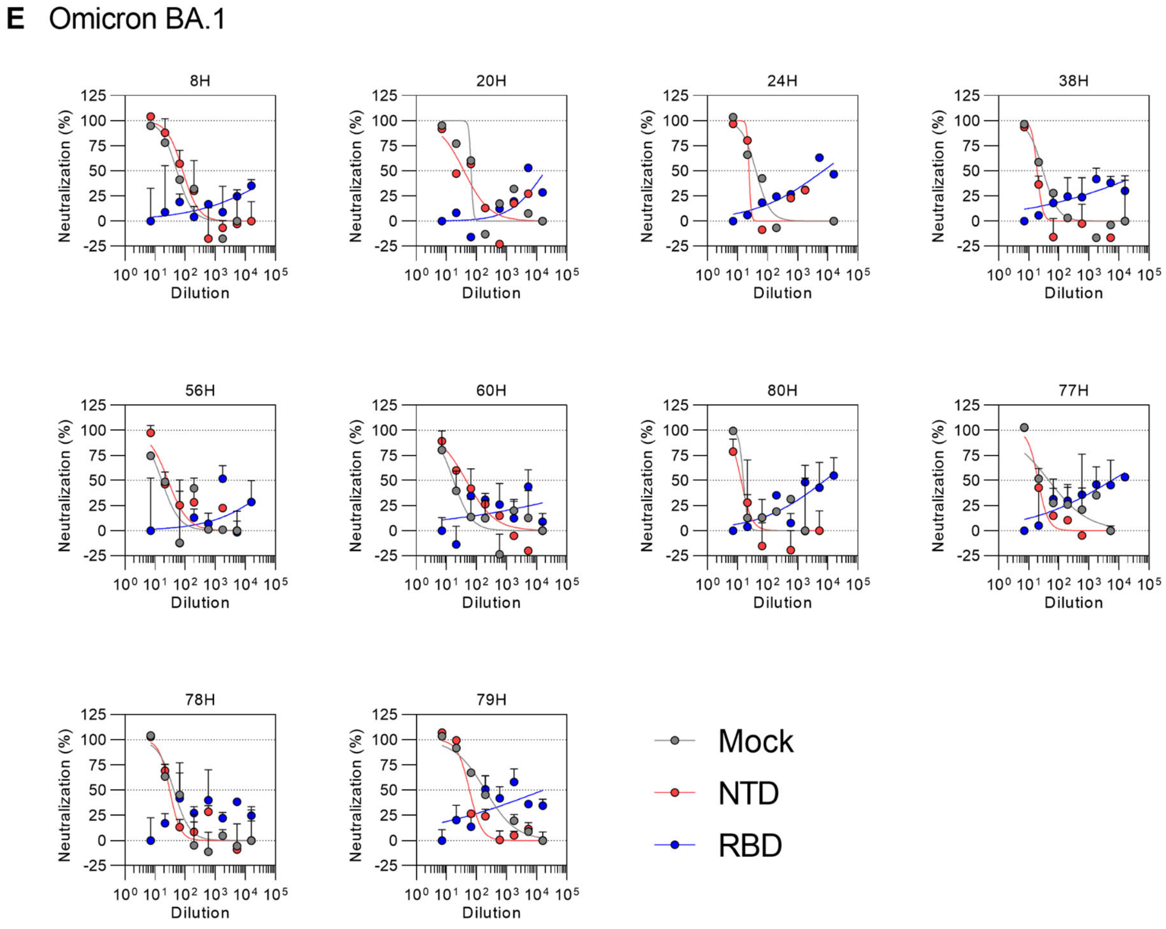

Figure S13. **Evaluation of vaccine-elicited plasma neutralizing activity against SARS-CoV-2 variants before and after antigen-specific depletion.** Normalized neutralization curves using SARS-CoV-2 Wuhan-Hu-1/G614 S, Alpha S, Beta S, Delta S, or Omicron BA.1 S VSV pseudoviruses using VeroE6-TMPRSS2 target cells following mock depletion or depletion with the Wuhan-Hu-1 RBD or NTD. Samples were collected from individuals vaccinated with two doses of mRNA-1273 or BNT162b12 and each color corresponds to one vaccinee. Individual points are the mean of duplicates and error bars (top only) are the standard deviation of those duplicates. Each graph is representative of at least two experimental replicates consisting of different batches of pseudoviruses. Patient demographics can be found in Table S2.

Table S1. CryoEM data collection and refinement statistics.

	Prefusion SARS-CoV-2 spike S_2 subunit<br>PDB 8DYA<br>EMD-27779
Data collection and processing	
Magnification	105,000
Voltage (kV)	300
Electron exposure ($e^{-}/Å^2$)	60
Defocus range (μm)	-0.5 - -2.5
Pixel size (Å)	0.843
Symmetry imposed	C3
Final particle images (no.)	137,737
Map resolution (Å)	3.7
FSC threshold	0.143
Map sharpening *B* factor ($Å^2$)	-161
Model building and refinement	
MolProbity score	1.23
Clashscore	1.82
Poor rotamers (%)	0.29
Ramachandran plot	
Favored (%)	95.80
Allowed (%)	3.68
Disallowed (%)	0.52

Table S2. Demographics data of enrolled plasma donors. The days since last vaccine dose for NVX-CoV2373 is unknown due to the double-blinded nature of the clinical trial. Subjects received their second vaccine dose on one date and their second placebo dose on another date. The time interval since last vaccine dose for both dates are listed.

Vaccine	Sample	Age	Sex	Days Since Symptom Onset or Last Vaccine Dose
1x Infected	10C	31	F	31
	114C	54	F	41
	13	54	M	32
	135C	43	M	49
	144C	30	M	48
	149C	25	M	40
	151	67	M	-
	151C	69	F	41
	157	42	F	-
	159	37	F	-
	182C	75	M	78
	200C	61	M	42
	21C	36	F	26
	235C	65	M	31
	224C	57	F	68
	28C	64	F	42
	30C	60	F	35
	32C	36	M	30
	54C	37	M	36
	57C	56	F	35

	60	36	M	-
	61	33	F	-
	65C	73	M	42
	73C	60	M	49
	75	24	F	-
	86	22	M	-
	98C	33	M	38
	9C	37	F	41
**2x mRNA-1273**	12H	42	F	10
	136040	40	M	20
	136049	26	F	18
	136052	47	M	21
	20H	33	F	6
	24H	24	M	7
	27H	59	M	11
	4H	23	M	6
	58H	50	F	15
	60H	42	F	8
	69H	68	F	13
	80H	35	F	50
	8H	79	M	11
	9H	75	F	7
**2x BNT162b2**	10H	33	M	14
	15H	52	M	9
	16H	46	F	6

	17H	76	M	17
	29H	64	M	12
	30H	61	F	10
	35H	60	M	33
	36H	37	M	9
	40H	38	F	13
	41H	36	F	13
	67H	40	F	13
	77H	28	F	14
	78H	22	M	15
	79H	27	F	20
**2x NXV-CoV2373**	6082.2	21	M	103
	6085.1	36	M	17 or 92
	6090.2	36	F	55
	6094.1	46	F	22 or 127
	6096.2	18	M	155
	6097.2	18	M	78
	6099.2	20	M	69 or 156
	6259.1	42	F	67
	6261.1	60	M	191
	6322.2	41	F	168
**2x Ad26.COV2.S**	0884-00033A00-001	38	M	14
	0884-00034J00-001	72	F	14
	0884-00036K00-001	43	F	12

	0884-0003WD00-001	54	M	13
	0889-0002SD00-001	43	M	14
	0889-0004FG00-001	28	F	16
	0889-0004KC00-001	36	F	13
	0889-0004WJ00-001	48	F	14
	0897-0001SB00-001	41	F	15
	0932-00064B00-001	60	F	13
	0933-0004FG00-001	51	M	14
	0933-0004KC00-001	26	F	14
**2x AZD1222**	38	29	F	30
	40	51	F	30
	41	39	F	30
	43	47	M	30
	45	48	F	30
	47	31	F	30
	48	37	F	30
	49	36	F	30
	51	29	F	30
	52	46	M	30
	53	37	F	30
	54	29	F	30
	56	45	F	30
	57	45	F	30
	58	28	F	30
	1	-	-	60-90

**2x Sputnik V**	10	-	-	60-90
	11	-	-	60-90
	12	-	-	60-90
	13	-	-	60-90
	14	-	-	60-90
	16	-	-	60-90
	3	-	-	60-90
	4	-	-	60-90
	5	-	-	60-90
	6	-	-	60-90
	7	-	-	60-90
	8	-	-	60-90
	9	-	-	60-90
**2x BBIBP-CorV**	1	47	F	79
	10	27	M	84
	11	28	F	102
	12	36	M	22
	13	26	F	102
	14	26	F	100
	16	39	M	97
	4	25	F	74
	5	26	F	71
	6	34	F	82
	7	29	M	76
	8	34	F	15

	9	33	F	15
**1x Infected, 2x BNT162b2**	10C	31	F	10
	114C	54	F	31
	144C	30	M	12
	149C	25	M	10
	151C	69	F	15
	182C	75	M	10
	28C	64	F	21
	30C	60	F	11
	54C	37	M	10
	65C	73	M	15
	73C	60	M	32
	9C	37	F	13
**1x Infected, 1x Ad26.COV2.S**	13	54	M	65
	135C	43	M	19
	200C	61	M	42
	21C	36	F	18
	224C	57	F	112
	32C	36	M	18
	57C	56	F	17
	98C	33	M	12
**1x Infected, 2x AZD1222**	61	33	F	30
	75	24	F	30
	86	22	M	30

Table S3. Analysis of statistical significance of differences observed for infection and vaccine-elicited antibody binding titers compared by vaccine, separated by antigen, and determined by Turkey's multiple comparisons test. *P < 0.05, **P < 0.01, ***P < 0.001, and ****P < 0.0001. ns: non-significant

S	mRNA-1273	BNT162b2	NVXCoV2373	Ad26COV2S	AZD1222	Sputnik V	BBIBP-CorV
Infected	****	****	ns	ns	***	ns	ns
mRNA-1273		ns	****	***	ns	****	***
BNT162b2			***	*	ns	**	*
NVXCoV2373				ns	ns	ns	ns
Ad26COV2S					ns	ns	ns
AZD1222						ns	ns
Sputnik V							ns
S_1	mRNA-1273	BNT162b2	NVXCoV2373	Ad26COV2S	AZD1222	Sputnik V	BBIBP-CorV
Infected	****	****	ns	*	****	ns	ns
mRNA-1273		ns	****	****	ns	****	****
BNT162b2			**	**	ns	***	***
NVXCoV2373				ns	ns	ns	ns
Ad26COV2S					ns	ns	ns
AZD1222						ns	ns
Sputnik V							ns
$S_{2(Pre)}$	mRNA-1273	BNT162b2	NVXCoV2373	Ad26COV2S	AZD1222	Sputnik V	BBIBP-CorV
Infected	**	ns	ns	ns	ns	ns	ns
mRNA-1273		ns	****	ns	*	***	ns
BNT162b2			****	ns	ns	*	ns
NVXCoV2373				ns	ns	ns	ns
Ad26COV2S					ns	ns	ns
AZD1222						ns	ns
Sputnik V							ns
$S_{2(Post)}$	mRNA-1273	BNT162b2	NVXCoV2373	Ad26COV2S	AZD1222	Sputnik V	BBIBP-CorV
Infected	ns	*	****	**	ns	ns	ns
mRNA-1273		ns	ns	ns	ns	ns	ns
BNT162b2			ns	ns	ns	ns	***
NVXCoV2373				ns	ns	ns	****
Ad26COV2S					ns	ns	****
AZD1222						ns	*
Sputnik V							*
NTD	mRNA-1273	BNT162b2	NVXCoV2373	Ad26COV2S	AZD1222	Sputnik V	BBIBP-CorV
Infected	****	****	ns	ns	**	ns	ns
mRNA-1273		ns	****	****	****	****	****
BNT162b2			****	****	**	****	****
NVXCoV2373				ns	ns	ns	ns
Ad26COV2S					ns	ns	ns
AZD1222						ns	ns
Sputnik V							ns
RBD	mRNA-1273	BNT162b2	NVXCoV2373	Ad26COV2S	AZD1222	Sputnik V	BBIBP-CorV
Infected	****	***	ns	ns	ns	ns	ns
mRNA-1273		ns	ns	***	*	****	****
BNT162b2			ns	*	ns	***	**
NVXCoV2373				ns	ns	*	ns
Ad26COV2S					ns	ns	ns
AZD1222						ns	ns
Sputnik V							ns

Table S4. Analysis of statistical significance of differences observed for infection and vaccine-elicited antibody binding titers compared by antigen, separated by vaccine, and determined by paired Turkey's multiple comparisons test. *P < 0.05, **P < 0.01, ***P < 0.001, and ****P < 0.0001. ns: non-significant

**Infected**	**S_1**	**$S_{2(Pre)}$**	**$S_{2(Post)}$**	**NTD**	**RBD**
**S**	****	ns	ns	****	****
**S_1**		****	****	****	****
**$S_{2(Pre)}$**			ns	****	****
**$S_{2(Post)}$**				****	****
**NTD**					ns
**mRNA-1273**	**S_1**	**$S_{2(Pre)}$**	**$S_{2(Post)}$**	**NTD**	**RBD**
**S**	ns	****	****	****	****
**S_1**		***	****	****	****
**$S_{2(Pre)}$**			****	ns	****
**$S_{2(Post)}$**				****	**
**NTD**					****
**BNT162b2**	**S_1**	**$S_{2(Pre)}$**	**$S_{2(Post)}$**	**NTD**	**RBD**
**S**	*	****	****	****	****
**S_1**		****	****	****	****
**$S_{2(Pre)}$**			****	**	****
**$S_{2(Post)}$**				***	ns
**NTD**					***
**NVX-CoV2373**	**S_1**	**$S_{2(Pre)}$**	**$S_{2(Post)}$**	**NTD**	**RBD**
**S**	ns	***	***	****	***
**S_1**		**	**	****	***
**$S_{2(Pre)}$**			ns	ns	ns
**$S_{2(Post)}$**				ns	ns
**NTD**					ns
**Ad26.COV2.S**	**S_1**	**$S_{2(Pre)}$**	**$S_{2(Post)}$**	**NTD**	**RBD**
**S**	**	**	****	****	****
**S_1**		ns	****	****	****
**$S_{2(Pre)}$**			****	****	****
**$S_{2(Post)}$**				ns	**
**NTD**					**
**AZD1222**	**S_1**	**$S_{2(Pre)}$**	**$S_{2(Post)}$**	**NTD**	**RBD**
**S**	ns	****	****	****	****
**S_1**		****	****	****	****
**$S_{2(Pre)}$**			ns	*	****
**$S_{2(Post)}$**				ns	***
**NTD**					**
**Sputnik V**	**S_1**	**$S_{2(Pre)}$**	**$S_{2(Post)}$**	**NTD**	**RBD**
**S**	**	**	**	****	****
**S_1**		ns	ns	****	****
**$S_{2(Pre)}$**			ns	*	***
**$S_{2(Post)}$**				ns	**
**NTD**					**
**BBIBP-CorV**	**S_1**	**$S_{2(Pre)}$**	**$S_{2(Post)}$**	**NTD**	**RBD**
**S**	***	*	ns	****	****
**S_1**		ns	ns	****	****
**$S_{2(Pre)}$**			ns	****	****
**$S_{2(Post)}$**				****	****
**NTD**					ns

Table S5. Analysis of statistical significance of differences observed for infection and vaccine-elicited antibody binding ratios compared by vaccine, separated by antigen, and determined by Turkey's multiple comparisons test. *P < 0.05, **P < 0.01, ***P < 0.001, and ****P < 0.0001. ns: non-significant

$S/S_{2(Post)}$	mRNA-1273	BNT162b2	NVXCoV2373	Ad26COV2S	AZD1222	Sputnik V	BBIBP-CorV
Infected	****	****	****	****	****	***	ns
mRNA-1273		ns	ns	ns	ns	ns	****
BNT162b2			ns	ns	*	**	****
NVXCoV2373				ns	ns	*	****
Ad26COV2S					ns	ns	****
AZD1222						ns	***
Sputnik V							**
$S_1/S_{2(Post)}$	mRNA-1273	BNT162b2	NVXCoV2373	Ad26COV2S	AZD1222	Sputnik V	BBIBP-CorV
Infected	****	****	****	****	****	****	ns
mRNA-1273		ns	ns	ns	ns	ns	****
BNT162b2			ns	ns	*	***	****
NVXCoV2373				ns	*	***	****
Ad26COV2S					ns	ns	****
AZD1222						ns	***
Sputnik V							*
$S_{2(Pre)}/S_{2(Post)}$	mRNA-1273	BNT162b2	NVXCoV2373	Ad26COV2S	AZD1222	Sputnik V	BBIBP-CorV
Infected	****	****	*	****	ns	ns	ns
mRNA-1273		*	ns	ns	*	ns	****
BNT162b2			***	ns	****	****	****
NVXCoV2373				ns	ns	ns	ns
Ad26COV2S					**	**	****
AZD1222						ns	ns
Sputnik V							ns

Table S6. Analysis of statistical significance of differences observed for infection and vaccine-elicited antibody binding titers compared by antigen, separated by vaccine, and determined by paired Turkey's multiple comparisons test. *P < 0.05, **P < 0.01, ***P < 0.001, and ****P < 0.0001. ns: non-significant

Infected, BNT162b2	S_1	$S_{2(Pre)}$	$S_{2(Post)}$
S	****	**	****
S_1		ns	****
$S_{2(Pre)}$			****
Infected, Ad26.COV2.S	S_1	$S_{2(Pre)}$	$S_{2(Post)}$
S	**	*	****
S_1		ns	***
$S_{2(Pre)}$			**
Infected, AZD1222	S_1	$S_{2(Pre)}$	$S_{2(Post)}$
S	ns	ns	ns
S_1		ns	ns
$S_{2(Pre)}$			ns

Table S7. Analysis of statistical significance of differences observed for antibody binding titers in previously infected individuals elicited before and after vaccination compared by antigen, separated by vaccine, and determined by paired Turkey's multiple comparisons test. *P < 0.05, **P < 0.01, ***P < 0.001, and ****P < 0.0001. ns: non-significant

Infected, 2x BNT162b2	
S	****
S_1	****
$S_{2(Pre)}$	****
$S_{2(Post)}$	*
**Infected, 1x Ad26.COV2.S**	
S	***
S_1	***
$S_{2(Pre)}$	***
$S_{2(Post)}$	ns
**Infected, 2x AZD1222**	
S	ns
S_1	ns
$S_{2(Pre)}$	ns
$S_{2(Post)}$	ns

Table S8. Analysis of statistical significance of differences observed for infection and vaccine-elicited antibody neutralizing titers. Determined by Turkey's multiple comparisons test. *P < 0.05, **P < 0.01, ***P < 0.001, and ****P < 0.0001. ns: non-significant

Neut.	mRNA-1273	BNT162b2	NVXCoV2373	Ad26COV2S	AZD1222	Sputnik V	BBIBP-CorV
Infected	****	*	ns	ns	ns	ns	ns
mRNA-1273		ns	*	**	**	***	**
BNT162b2			ns	ns	ns	ns	ns
NVXCoV2373				ns	ns	ns	ns
Ad26COV2S					ns	ns	ns
AZD1222						ns	ns
Sputnik V							ns

Table S9. Analysis of statistical significance of differences observed for infection and vaccine-elicited antibody neutralizing titers in previously infected individuals. Determined by Turkey's multiple comparisons test. *P < 0.05, **P < 0.01, ***P < 0.001, and ****P < 0.0001. ns: non-significant

Neutralization	Ad26.COV2.S	AZD1222
BNT162b2	*	ns
Ad26.COV2.S		ns
